# The early impact of the COVID-19 pandemic on patients with severe mental illness: An interrupted time-series study in South-East England

**DOI:** 10.1192/j.eurpsy.2022.22

**Published:** 2022-05-18

**Authors:** Ed Penington, Belinda Lennox, Galit Geulayov, Keith Hawton, Apostolos Tsiachristas

**Affiliations:** 1 Nuffield Department of Population Health, University of Oxford, Oxford, United Kingdom; 2 Department of Psychiatry, University of Oxford, Oxford, United Kingdom

**Keywords:** Severe mental illness, COVID-19, Interrupted time series, Mental health services, Health of the Nation Outcome Scales (HoNOS)

## Abstract

**Background:**

Deterioration in general population mental health since the start of the COVID-19 pandemic has been reported, but the impact of the pandemic on people with severe mental illness (SMI) has received less attention.

**Aims:**

To understand the impact of the early stages of the pandemic on the patients with SMI, in terms of provision of mental health care and patient outcomes.

**Method:**

We examined records of 34,446 patients with SMI in Oxford Health Foundation Trust between March 2016 and July 2020. We used interrupted time-series analysis to estimate the immediate and subsequent changes in weekly rates of the use of community mental health services, hospitalization, and patient outcomes (as measured by Health of the Nation Outcome Scales, or HoNOS, scores) during the weeks of lockdown between March 23, 2020 and July 3, 2020.

**Results:**

Mean total HoNOS scores for all patients deteriorated in the weeks subsequent to lockdown (0.060 per week; 95%CI: 0.033, 0.087). Scores for patients with a history of psychosis deteriorated immediately (0.63; 95% CI: 0.26, 1.0). There was an immediate decrease in weekly referrals to community and outpatient services (−196; 95%CI: −300, −91) and no immediate change in weekly inpatient admissions (−4.2; 95%CI: −9.9, 1.5) or weekly total contacts (−26; 95%CI: −475, 423).

**Conclusions:**

Patients with SMI were negatively impacted during the early stages of the COVID-19 pandemic. Patients with a history of psychosis experienced distinct and immediate impacts. During the same period, referrals to community and outpatient services fell with no consequent impact on inpatient admissions.

## Research in Context

### Evidence before this study

We conducted a review of existing evidence on the impact of COVID-19 on patients with severe mental illness. We searched for studies with terms related to both SMI (“mental illness” or “psychosis”) and COVID-19 (“COVID” or “coronavirus”) in PubMed and Google Scholar. We also reviewed citations of relevant papers (“snowballing”) and the contents of selected psychiatric journals in the period following the onset of the pandemic.

An increase in the prevalence of mental illness—particularly depression and anxiety—in the general populations of multiple countries since the start of the pandemic has been well documented. Despite this, early reports generally indicated a decline in demand for psychiatric emergency care. UK-based studies found significant reductions in recorded primary care contacts for patients with severe mental illness, and no clear change in patient demand following a large-scale transition to remote secondary care. Multiple studies found that aggregate levels of self-harm and suicide have not increased as a result of the pandemic. Research on how the pandemic affected people with pre-existing mental health conditions has been limited and mainly focused on psychiatric resource use or self-report surveys.

### Added value of this study

To our knowledge, this study is the first to show clear deterioration in the well-being and functioning of patients with SMI, as measured by routinely recorded clinical measures. We found that this deterioration has been gradual and is not restricted to any particular measure of well-being, symptoms, or social functioning. We also found evidence of distinct, acute impacts on patients with a recorded history of psychosis.

### Implications of all the available evidence

Policy-makers and researchers considering the impact of the COVID-19 and future pandemics on mental health should take into account the distinct experiences of patients with SMI. The potential unmet need for early psychiatric care may increase demand for more intensive care in future. Service providers should budget with this follow-on effect in mind and plan to ensure continuity of access in future pandemics. This should prompt further research into how mental health services can best prepare patients with mental illness for societal and personal disruptions.

## Introduction

As the COVID-19 pandemic spread across the world in the early months of 2020, so too did a wave of public health restrictions aimed at limiting the spread of the virus, including lockdowns, the closure of schools and businesses, bans on public gatherings, travel restrictions, and rapid changes in healthcare provision [1]. Early commentaries [2, 3] warned of potential increase in suicides and self-harm as risk factors such as social isolation and economic hardship were exacerbated, together with particular impacts on people with mental health disorders, especially as they may be more susceptible to infection and the emotional stress of the pandemic [4]. Individuals with mental disorders have experienced a higher risk of hospitalization and mortality due to COVID-19 [5] and people diagnosed with COVID-19 have in turn been found to have higher risk of neurological and mental disorders [6]. While there has been extensive research on the mental health of the general population (primarily through surveys) and the prevalence of self-harm [7, 8] and suicides [9], research on outcomes for those with severe mental illness (SMI) has been more limited. A number of studies have conducted surveys of self-reported outcomes for patients with SMI [10, 11]; however, these methods struggle to capture the immediate impact of the pandemic.

The aims of this study were therefore twofold: firstly, to assess whether the early stages of the pandemic impacted on the well-being and social functioning of patients with SMI; secondly, to understand how the early stages of the pandemic had impacted the provision and use of secondary mental health services.

## Methods

### Data and variables of interest

We obtained access to electronic patient records for the Oxford Health NHS Foundation Trust (OHFT), which provides National Health Service (NHS) funded secondary care for patients with mental illness in an area of South East England (mainly the counties of Oxfordshire and Buckinghamshire). Care provided by OHFT includes inpatient psychiatric care, Community Mental Health Teams (CMHT), Early Intervention in Psychosis (EIP) services, Crisis resolution and home Treatment (CT) teams, and inpatient Psychiatric Liaison (PL) teams. The data only covered care provided by OHFT directly, excluding private or out-of-area care, as well as care provided by third sector, primary care, and some talking therapies covered by the NHS [12].

The sample consisted of 34,446 patients with any care contact between March 2016 and July 2020, the majority of whom (*N* = 31,731; 92.1%) were referred to OHFT services before the pandemic began. Records for the sample included referrals to and contacts with community and outpatient mental health services, psychiatric hospital admissions, and the Health of the Nation Outcome Scales [13] (HoNOS) assessments, which is a clinician recorded health measurement routinely collected for patients of OHFT. HoNOS consists of 12 scales of health and social functioning for which values are recorded ranging between 0 (no problem) and 4 (severe to very severe problem). Each of the 12 scales is listed in Supplementary Appendix 2.3.

These records were transformed to weekly totals and weekly means of outcomes of the patients assessed or treated. For the purposes of this analysis, we defined the following variables of interest:

- *HoNOS assessments*: total number of HoNOS assessments; number of HoNOS assessments specifically designated as “initial,” “ongoing,” or “discharge.”
- *HoNOS scores:* mean weekly total score (out of a maximum possible of 48); for each subscale, the percentage of assessments reporting any problem (score 1 or higher); and the mean overall score (out of a maximum of 4).
- *HoNOS follow-ups:* statistics for the subset of HoNOS assessments that were performed within 12 weeks of a prior assessment for the same patient, specifically mean change in score; percentage reporting an overall deterioration (higher total score); and percentage reporting an overall improvement (lower total score).
- *Community and outpatient referrals*: total weekly count of unique patients referred to any service; weekly count of patients referred to any service for the first time; and weekly number of referrals to specific types of services: EIP, CMHT, Crisis Teams (CT) and PL.
- *Community and outpatient access*: mean weekly time (in days) between referral and first contact with a service; and proportion of referrals that result in a contact within 30 days.
- *Community and outpatient contacts*: total number of contacts marked as attended by patients in the OHFT diary system; number of attended first contacts (i.e., no prior recorded contact within the study period); number of attended face-to-face contacts; number of attended remote (i.e., anything other than face-to-face) contacts; and number of attended contacts (either remote or face-to-face) with specific types of community and outpatient services: EIP, CMHT, CT, and PL.
- *Inpatient admissions*: total number of new (i.e., not an internal transfer) inpatient admissions; number of first inpatient admissions (i.e., no prior recorded inpatient episode within the study period); admissions to acute or psychiatric intensive care unit wards specifically (mainly excluding admission of older adults for dementia and related conditions); percentage of admissions that were readmissions (i.e., patients had been discharged within the last 30 days); total inpatient bed days; count of unique patients who were in an inpatient setting at any point in a week; and mean length of inpatient stays (in days).

Ethical approval was not required for this study as it was conducted on de-identified, routinely collected data. The data for this research were accessed through the Clinical Record Interactive Search (CRIS) system, which contains pseudonymized records of mental health care provided by a number of NHS trusts across England. All applications to access the CRIS database are reviewed by the CRIS Oversight Group.

### Statistical analysis

For each weekly variable, we conducted a single-unit interrupted time-series analysis (ITSA) to estimate the immediate and subsequent impact of COVID-19 and associated public health measures. ITSA models the impact of an intervention (in this case the COVID-19 pandemic) on a time-varying outcome. This approach is considered a strong quasi-experimental design, especially in contexts where control observations are not available, and has been applied across a wide range of healthcare settings [14]. The ITSA estimates the “immediate” change, which shows the shift in estimated value from the last week before the interruption to the first week after it and the “subsequent” change in weekly trend following the interruption. This distinction reflects the fact that the COVID-19 pandemic may have had both instantaneous effects (e.g., a switch to remote provision) and cumulative effects (e.g., increasing strain on the healthcare system or longer periods of time in social isolation). The “combined” impact shows the overall change in the variable after both the immediate change and 15 weeks of subsequent change, indicating the difference in the outcome at the end of the lockdown period (July 3, 2020) relative to pre-COVID-19 trends. The relative value of each coefficient is expressed as a percentage of the predicted value for the week commencing March 16, 2020, that is, the average in the week immediately preceding lockdown.

Pre-existing time trends, immediate impact, and subsequent impact were initially estimated with ordinary least squares (OLS). We tested for serial correlation using the Ljung–Box test. For outcome variables where the test showed the presence of serial correlation in the residuals of the OLS model, an auto-regressive integrated moving average (ARIMA) model was fitted. This model controls directly for serial correlation (through AR and MA terms), nonstationarity (through differencing), and seasonal trends (through seasonal differencing), ensuring unbiased estimates of the immediate and subsequent changes caused by the interruption [15]. The order of auto-regressive, integration, and moving average terms for each outcome was selected algorithmically by step-wise comparisons of goodness-of-fit as measured by the Akaike information criterion, in line with the literature [15] (for more details, see Supplementary Appendix 3.2). The final ARIMA form (expressed as AR, I, MA) for each outcome is listed with the full results of the ITSA in Supplementary Appendix 1. In practice, none of the selected models required differencing or seasonal terms. All predicted values, including those used for the relative scaling of coefficients, are in their structural form, meaning the ARIMA elements used in fitting the model are excluded.

Variables measuring mean outcomes or durations for patients assessed, admitted, or referred in a given week were likely to be influenced not only by the effects of COVID-19 but also by the changes in patient characteristics. To correct for this, a propensity score representing the likelihood of a week occurring after the lockdown date was estimated based on gender, ethnicity, deprivation, and ICD-10 diagnosis codes. The propensity score was used as both an inverse weight and a control variable in the relevant regressions (for more details, see Supplementary Appendix 3.3). In addition, data covering mean durations (inpatient length of stay and mean time from referral to contact) were downwardly biased due to ongoing episodes at the end of the sample period. To correct for this, the expected values of censored observations were imputed with parametric survival models (for more details, see Supplementary Appendix 3.1). Predicted values for these variables are presented for the population of patients in question (assessed or admitted) in the given week.

Some outcome variables are counts describing the number of individuals or events in a given week, such as inpatient admissions or recorded contexts. While it may be more appropriate to model these variables with count distributions, this would not have allowed consistent and comparable use of our OLS and ARIMA specifications (which use a normal distribution) across all outcome variables. Since the normal distribution approximates the binomial distribution at sufficiently high numbers, we tested each count variable for systematic differences in goodness of fit between simple OLS and count regressions. Where count models outperformed OLS, the variable was excluded from results; no outcome variables for the full population were excluded by this process. For more details on this procedure, see Supplementary Appendix 3.4.

### Robustness of the results

The exact date of the imposition of COVID-19 restrictions was assumed to be March 23, but awareness of the virus and its potential impacts on hospitals had been growing for months prior to this date, with some organizations taking proactive measures earlier in March. To account for the potential inclusion of some COVID-19 effects in the pre-COVID-19 period, all ITSA models were also run with identical specifications but with the weeks from January 2020 to March 2020 removed. Full results for this analysis are provided in Supplementary Appendix 1.2.

### Subgroup analysis

The analysis was also run on the subpopulations of patients who have been recorded living in a deprived area (bottom quintile of the Index of Multiple Deprivation 2019) and patients with any recorded diagnosis of psychosis (as indicated by recorded ICD-10 codes). Results for inpatient admissions, HoNOS follow-ups, and referrals to and contacts with specific services for these subpopulations have not been included as the normal approximation of count variables does not hold in the smaller samples. Full results for these analyses are provided in Supplementary Appendices 1.3 and 1.4.

### Role of the funding source

This work was funded by University of Oxford Medical Sciences Division Covid Response Fund. The Funder had no role in the research undertaken.

## Results

### Patient sociodemographic and clinical characteristics

Descriptive statistics of the patients in our sample are shown in Table 1. Just over half of individuals in the sample (55.4%) were female. Only a small proportion of individuals with an ethnicity recorded (7.5%) were from a Black or other minority ethnic background. The mean age at first referral within the study period was 41.2. The most common recorded diagnoses were for depressive disorders (14.0%) and psychosis (11.2%). However, these are likely to be underestimates of the true prevalence of these disorders in our sample due to inconsistent use of the ICD-10 field (only 48.3% of patients having a diagnosis recorded).

**Table 1.** Descriptive statistics.

Referred to any OHFT service (*n*)	100.0% (34,446)
% patients with first referral before COVID-19 (*n*)	92.1% (31,731)
Mean age at first referral (sd, *n*)	41.2 (20.7, 33,701)
Female % (*n*)	55.4% (18,658)
Black or minority ethnic % (*n*)	7.5% (1,601)
Residence in top 20% most deprived areas % (*n*)	8.8% (2,938)
Recorded diagnosis of psychosis (*n*)	11.2% (3,872)
Recorded diagnosis of depressive disorder (*n*)	14.0% (4,818)
Recorded diagnosis of schizophrenia or schizotypal disorder (*n*)	8.7% (2,980)
Recorded diagnosis of bipolar disorder (*n*)	4.9% (1,702)
Recorded diagnosis of personality disorder (*n*)	7.3% (2,517)
Recorded diagnosis of dementia (*n*)	7.3% (2,504)
Recorded diagnosis of anxiety disorders (*n*)	6.4% (2,190)
Recorded diagnosis of stress disorders (*n*)	3.6% (1,244)
Recorded diagnosis of eating disorder (*n*)	2.0% (688)
Death recorded (*n*)	7.5% (2,600)
Referred to EIP services (*n*)	5.7% (1,972)
Referred to Community Mental Health Teams (*n*)	30.3% (10,453)
Referred to Crisis Teams (*n*)	11.2% (3,873)
Referred to Psychiatric Liaison services (*n*)	16.4% (5,661)
Mean community/outpatient referrals (sd, *n*)	3.1 (3.3, 106,509)
Mean scheduled contacts (sd, *n*)	17.0 (33.8, 586,651)
Any recorded inpatient episode	7.5% (2,588)
Mean inpatient episodes (sd, *n*)	1.4 (1.1, 3,707)
Mean inpatient bed days (sd, *n*)	103.5 (2,290, 267,877)
Any HoNOS assessment (*n*)	72.1% (24,828)
Mean HoNOS assessments (sd, *n*)	1.5 (1.7, 51,880)
Mean total HoNOS score (sd, *n*)	11.6 (6.3, 51,880)

*Note:* Reported percentages may not use the full number of patients in the sample as the denominator due to incomplete reporting for some characteristics.

Abbreviations: EIP, Early Intervention in Psychosis; HONOS, Health of the Nation Outcome Scales; OHFT, Oxford Health NHS Foundation Trust.

Analysis of the national representativeness of our sample was not straightforward as demographic and prevalence data are not collected for the OHFT geographical area. However, we compared OHFT’s service statistics (as collected in the Mental Health Bulletin Annual Report, 2019–2020 [16]) and the prevalence statistics of OHFT’s partner Clinical Commissioning Groups (as collected in the Qualities and Outcomes Framework, 2020–2021 [17]) to national statistics. In general, OHFT is a slightly larger than the average NHS mental health trust, serving areas with nationally representative prevalence of mental and neurological disorders (full comparisons are shown in Supplementary Appendix 2.1). Our sample was less deprived than the population of England (8.8% of people residing in the most deprived 20% of areas), but this may be biased by the poorer collection of residence records for more deprived patients.

**Figure 1.**
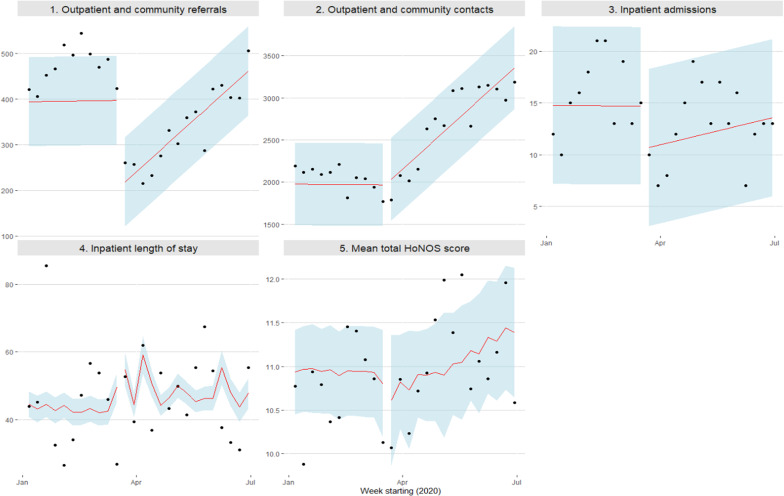
Interrupted time-series graphs of key outcomes.

The coefficients of interest for the main set of ITSA models are shown in Table 2, while full result tables (including prepandemic constant, weekly trend and ARIMA specification) are shown in Supplementary Appendix 1.1. Summary statistics of the weekly aggregate outcomes are shown in Supplementary Appendix 2.2. Figure 1 shows the pre- and postpandemic trend of predicted values for key outcome variables during 2020, based on the main set of models estimated using data from the entire study period.

**Table 2.** Interrupted time-series analysis results.

Outcomes		Immediate	Subsequent	Combined
*Outpatient and community referrals:*		Coefficient (95% CI) [relative change]	Coefficient (95% CI) [relative change]	Linear combination after 15 weeks (95% CI) [relative change]
	Total referrals	−195.6*** (−300.0, −91.2) [−49.2%]	17*** (7.4, 26.7) [4.3%]	60 (−8.3, 128.3) [15.1%]
	First referrals	−50.6 (−117.7, 16.5) [−52.6%]	3.3 (−2.9, 9.6) [3.5%]	−0.5 (−41.5, 40.6) [−0.5%]
	Early intervention in psychosis	−2.11 (−8.22, 4.00) [−27.6%]	0.26 (−0.38, 0.90) [3.4%]	1.78 (−3.31, 6.87) [23.2%]
	Community mental health teams	−54.78*** (−84.64, −24.92) [−88.4%]	5.61*** (3.11, 8.10) [9%]	29.3 (−1.39, 59.98) [47.3%]
	Crisis Teams	−23.47* (−44.56, −2.39) [−68.6%]	2.29** (0.66, 3.91) [6.7%]	10.83 (−0.08, 21.74) [31.7%]
	Psychiatric Liaison	−21.2 (−53.26, 10.85) [−42.7%]	1.47 (−1.33, 4.27) [3%]	0.83 (−15.69, 17.34) [1.7%]
	Mean days to contact after referral	−20 (−49.0, 8.9) [−19.9%]	−1.7 (−5.4, 1.9) [−1.7%]	−46.1* (−83.5, −8.8) [−45.7%]
*Outpatient and community contacts*:				
	Total contacts	−26.2 (−475.5, 423.2) [−1.3%]	95.1*** (46.2, 144.0) [4.8%]	1,400.2*** (997.5, 1,802.8) [71.2%]
	First contacts	−10.4 (−46.8, 26.1) [−10.5%]	0.5 (−2.8, 3.8) [0.5%]	−2.8 (−25.5, 19.9) [−2.8%]
	Face-to-face contacts	−1,209.3*** (−1,695.0, −723.6) [−74.9%]	16 (−94.8, 126.7) [1%]	−969.8 (−2,386.3, 446.6) [−60.1%]
	Remote contacts	917.3*** (838.1, 996.6) [231%]	85.6*** (69.9, 101.3) [21.6%]	2,201.2*** (1,989.3, 2,413.0) [554.3%]
	Early Intervention in Psychosis	3 (−69.6, 75.6) [1.9%]	8.4* (0.6, 16.2) [5.4%]	128.9*** (56.6, 201.2) [82.6%]
	Community Mental Health Teams	129.3** (38.3, 220.3) [21.6%]	19.8*** (9.1, 30.5) [3.3%]	426.3*** (321.8, 530.8) [71.2%]
	Crisis Teams	−26.1* (−49.0, −3.2) [−66%]	2.2** (0.6, 3.8) [5.5%]	6.5 (−6.2, 19.2) [16.5%]
	Psychiatric Liaison	−20.3 (−55.9, 15.3) [−36.2%]	1.5 (−2.0, 5.0) [2.7%]	2 (−23.1, 27.1) [3.6%]
*Inpatient admissions:*				
	Total admissions	−4.2 (−9.89, 1.49) [−28.6%]	0.21 (−0.41, 0.83) [1.4%]	−1.03 (−6.85, 4.79) [−7%]
	First admissions	−3.19 (−6.71, 0.34) [−32%]	0.14 (−0.23, 0.51) [1.4%]	−1.09 (−4.42, 2.24) [−11%]
	Admissions to acute/psychiatric intensive care unit wards	−3.39 (−7.62, 0.84) [−32.9%]	0.19 (−0.29, 0.66) [1.8%]	−0.61 (−5.08, 3.86) [−5.9%]
	% who were discharged in last 30 days	−2.58* (−4.97, −0.19)	0.03 (−0.20, 0.26)	−2.13 (−4.96, 0.7)
	Total bed days	−1.7 (−54.0, 50.5) [−0.2%]	0.2 (−12.2, 12.6) [0%]	0.9 (−185.5, 187.3) [0.1%]
	Inpatient length of stay	3.4 (−7.8, 14.5) [6.8%]	0.1 (−1.0, 1.2) [0.2%]	4.6 (−8.6, 17.9) [9.4%]
*HoNOS assessments:*				
	Total assessments	−30.78 (−81.38, 19.81) [−15.5%]	0.89 (−5.20, 6.98) [0.5%]	−17.39 (−69, 34.22) [−8.8%]
	Initial assessments	−21.51 (−50.42, 7.40) [−19.4%]	0.33 (−2.88, 3.53) [0.3%]	−16.62 (−42.37, 9.13) [−15%]
*HoNOS scores:*				
	Mean total score	−0.21 (−0.44, 0.02)	0.06*** (0.03, 0.09)	0.69*** (0.37, 1.00)
*HoNOS repeated assessments (within 12 weeks)*				
	Mean score change	−1.727*** (−2.101, −1.353)	0.352*** (0.322, 0.381)	3.547*** (3.180, 3.914)
	% with overall deterioration	−3.78** (−6.04, −1.53)	0.67*** (0.49, 0.85)	6.23*** (4.02, 8.44)
	% with overall improvement	−0.85 (−3.06, 1.35)	−0.23** (−0.41, −0.06)	−4.34*** (−6.49, −2.18)

*Note:* Relative values [%] for volume and duration variables are reported as a percentage of the predicted value for the week of March 16, 2020 (i.e., immediately preceding lockdown).

* *p*-value < 0.05;

** *p*-value < 0.01;

*** *p*-value < 0.001.

### Health outcomes

There was a gradual deterioration (increase) in mean total HoNOS scores at a rate of 0.06 per week (95% CI: 0.03, 0.09), leading to a combined increase of 0.69 by the end of the study period (95% CI: 0.37, 1.00). This was driven by deterioration in: agitated behavior (0.015; 95% CI: 0.010, 0.019); self-injury (0.005; 95% CI: 0.001, 0.009); problem-drinking or drug-taking (0.008; 95% CI: 0.004, 0.011); cognitive problems (0.012; 95% CI: 0.007, 0.017); physical illness (0.012; 95% CI: 0.006, 0.018); other mental and behavioral problems (0.007; 95% CI: 0.002, 0.012); problems with activities of daily living (0.009; 95% CI: 0.004, 0.013); and problems with occupation and activities (0.007; 95% CI: 0.003, 0.012).

The following subscales showed a combined increase above prepandemic trends at the end of the study period: agitated behavior (0.133; 95% CI: 0.080, 0.186); problems with occupation and activities (0.125; 95% CI: 0.073, 0.177); problems with activities of daily living (0.114; 95% CI: 0.064, 0.163); cognitive problems (0.088; 95% CI: 0.027, 0.149); physical illness (0.084; 95% CI: 0.045, 0.123); and hallucinations and delusions (0.080; 95% CI: 0.028, 0.132).

Two subscales showed subsequent improvement following an immediate deterioration: problems with relationships (−0.014; 95% CI: −0.019, −0.009) and problems with living conditions (−0.005; 95% CI: −0.009, −0.001). Both ended the study period with a statistically insignificant changes of (−0.058; 95% CI: −0.121, 0.005) and (−0.001; 95% CI:-0.045, 0.044), respectively.

HoNOS assessments undertaken within 12 weeks of a prior assessment show a similar pattern, with an immediate improvement in the mean total change in scores between assessments (−1.7; 95% CI: −2.1, −1.4) and a subsequent deterioration (0.35 per week; 95% CI: 0.32, 0.38), with a combined increase in the mean change in scores between assessments of 3.55 (95% CI: 3.18, 3.91) by the end of the study period. There was a combined 6.23 percentage point increase in reassessments recording overall deterioration (95% CI: 4.02, 8.44) and a 4.34 p.p. reduction in reassessments reporting overall improvement (95% CI: −6.49, −2.18).

No significant changes were detected in the number or type of HoNOS assessments conducted by clinicians during the COVID-19 period. Full results for ITSA analysis of HoNOS subscales are reported in Supplementary Appendix 1.1 for both the mean score for all assessments (indicating overall severity) and the proportion of assessments with any score greater than 0 (indicating the prevalence of any problems).

### Use of mental health services

Weekly outpatient and community referrals fell immediately on the imposition of COVID-19 lockdown, by −195.6 (95% CI: −300, −91.2), equivalent to 49.2% of the predicted rate for the week immediately preceding lockdown. Referrals then recovered over the lockdown period at a rate of 17.0 per week (95% CI: 7.4, 26.7), leaving a final combined change that was not statistically significant (60.0; 95% CI: −8.3, 128.3). The reduction in referrals was most pronounced for Crisis Teams (−23.5; 95% CI: −44.6, −2.4) and CMHT (−54.8; 95% CI: −84.64, −24.92), both of which recovered over the subsequent weeks at rates of 2.3 per week (95% CI: 0.7, 3.9) and 5.6 per week (95% CI: 3.1, 8.1), respectively.

The number of weekly face-to-face community and outpatient contacts fell immediately by −1,209 (95% CI: −1,695,−723), equivalent to 70.4% of the pre-2020 mean of 1,716 weekly face-to-face contacts, while remote contacts increased by 917 per week (95% CI: 838, 997). There was therefore an instantaneous switch in the method of community and outpatient contacts from predominantly face-to-face to predominantly remote, but no overall change in the weekly rate of contacts recorded due to the COVID-19 lockdown (−26 per week; 95% CI: −476; 423). In fact, the total number of weekly contacts grew gradually over the subsequent weeks at a rate of 95 per week (95% CI: 46, 144), leading to a combined increase of 1,400 contacts per week (95% CI: 998, 1,803) by the start of July 2020. The only service to see a reduction in the rate of contacts was Crisis Teams, with contacts decreasing by −26 per week (95% CI: −49, −3.2), but this recovered over the lockdown period at a rate of 2.2 per week (95% CI: 0.6, 3.8) to a combined change that was not statistically significant (6.5; 95% CI: −6.2, 19.2).

The total weekly number of recorded inpatient admissions did not change by a statistically significant amount (−4.2; 95% CI: −9.9, 1.5). No change in the severity of admissions (as measured by mean length of stay) was detected (3.4; 95% CI: −7.8, 14.5). The proportion of admissions that were readmissions (i.e., had been discharged in the previous 30 days) reduced by −2.6 percentage points (95% CI: −5.0, 0.2).

### Sensitivity analysis and robustness

The exclusion of all weeks in 2020 prior to the first lockdown in England from the analysis resulted in almost identical coefficients. However, the immediate and subsequent changes for contacts and referrals differ when the early weeks of 2020 are excluded, suggesting the impact of lockdown on service provision did not occur precisely at the time of lockdown (e.g., through measures put in place in anticipation of lockdown).

### Results from the subgroup analysis

The subgroups on which our analysis was also conducted represent small proportions of the overall sample: 8.8% of patients (2,938) resided in the top 20% most deprived areas and 11.2% of patients (3,872) had a recorded diagnosis of psychosis. Due to limited recording of addresses and diagnoses, these subgroups will not include all patients in the sample who have lived in deprived areas or have a history of diagnosis. Patients are more likely to have their diagnosis or address formally recorded through more frequent contacts with services, meaning outcomes for these groups are likely to be reflective of, but more severe than, the full population of patients from deprived areas or with a history of psychosis. Summary results of the subgroup analysis are shown in Table 3.

**Table 3.** Sub-group interrupted time-series analysis results.

		Residence in a deprived area			Recorded diagnosis of psychosis		
Outcomes		Immediate	Subsequent	Combined	Immediate	Subsequent	Combined
*Outpatient and community referrals*:		Coefficient (95% CI)	Coefficient (95% CI)	Linear combination after 15 weeks (95% CI)	Coefficient (95% CI)	Coefficient (95% CI)	Linear combination after 15 weeks (95% CI)
	Total referrals	−16.8** (−28.9, −4.8)	1.9*** (0.8, 3.1)	12.1* (2.7, 21.6)	−10.8 (−25, 3.4)	1.0 (−0.9, 2.8)	3.6 (−14.4, 21.6)
	First referrals	−4.5** (−7.4, −1.7)	0.3* (0, 0.6)	0.2 (−2.5, 2.9)	1.8 (−0.8, 4.3)	−0.1 (−0.4, 0.2)	0.4 (−2, 2.9)
	Mean days to contact after referral	−31.4*** (−48.3, −14.4)	−0.8 (−1.9, 0.3)	−43*** (−58.3, −27.8)	−16.9*** (−25.5, −8.3)	−2.3*** (−3.5, −1.2)	−52.1*** (−68.5, −35.7)
*Outpatient and community contacts*:							
	Total contacts	−15.8 (−87.4, 55.7)	11.1** (4.1, 18.1)	150.9*** (101.4, 200.3)	48.2 (−99.9, 196.3)	26.0** (10.5, 41.5)	438.2*** (314.2, 562.2)
	First contacts	−0.6 (−4.1, 2.9)	0.0 (−0.3, 0.4)	0.0 (−3.3, 3.2)	1.2 (−1.4, 3.7)	−0.1 (−0.3, 0.2)	−0.1 (−2.5, 2.3)
	Face-to-face contacts	−98.6 (−204.3, 7.1)	2.3 (−6.4, 11.1)	−63.5* (−118.6, −8.3)	−362.8** (−600.7, −124.9)	8.4 (−22.2, 38.9)	−237.1 (−549.6, 75.5)
	Remote contacts	79.9*** (68.2, 91.7)	8.7*** (7.5, 10.0)	210.6*** (197.8, 223.3)	397.6*** (378.7, 416.6)	18.4*** (16.4, 20.4)	673.6*** (655.6, 691.6)
*Inpatient admissions*:							
	Total bed days	−7.1 (−43.6, 29.4)	−0.8 (−6.5, 4.9)	−18.8 (−100.7, 63.1)	24.8 (−118.2, 167.7)	−1.7 (−14, 10.7)	−0.1 (−135.9, 135.6)
*HoNOS assessments:*							
	Total assessments	−4.92 (−10.12, 0.28)	0.51 (−0.05, 1.06)	2.72 (−2.06, 7.5)	0.19 (−12.3, 12.68)	0.05 (−1.35, 1.44)	0.89 (−10.94, 12.72)
	Initial assessments	−2.77 (−6.41, 0.88)	0.22 (−0.17, 0.61)	0.51 (−2.84, 3.86)	0.54 (−3.46, 4.54)	−0.06 (−0.48, 0.37)	−0.31 (−3.98, 3.36)
*HoNOS scores:*							
	Mean total score	−2.5*** (−3.22, −1.79)	0.18*** (0.12, 0.24)	0.19 (−0.5, 0.87)	0.63*** (0.26, 1.0)	0.01 (−0.02, 0.04)	0.76*** (0.36, 1.15)

* *p*-value < 0.05;

** *p*-value < 0.01;

*** *p*-value < 0.001.

Patients with a residence recorded in the most deprived areas experienced an immediate, statistically significant improvement in mean total HoNOS scores (−2.50; 95% CI: −3.22, −1.79). Scores subsequently deteriorated at a rate of 0.18 per week (95% CI: 0.12, 0.24), leading to a combined change that was not statistically significant (0.19; 95% CI: −0.50, 0.87). Full results for this subgroup are shown in Supplementary Appendix 1.3.

Patients with a recorded history of psychosis saw an immediate deterioration in mean total HoNOS scores (0.63; 95% CI: 0.26, 1.0). Combined with a statistically insignificant subsequent further deterioration in scores (0.01; 95% CI: −0.02, 0.04), mean total scores were estimated to be 0.76 higher for this group by the end of the study period (95% CI: 0.36, 1.15). Both the immediate and subsequent changes in total score are composed of many countervailing movements within specific subscales, full results for which are shown in Supplementary Appendix 1.4. Notably, the following subscales showed statistically significant combined increases at the end of the study period: self-injury (0.198; 95% CI: 0.149, 0.247); problem-drinking or drug-taking (0.172; 95% CI: 0.118, 0.226); physical illness (0.172; 95% CI: 0.120, 0.224); cognitive problems (0.160; 95% CI: 0.095, 0.224); problems with occupation and activities (0.119; 95% CI: 0.051, 0.186); and depressed mood (0.079, 95% CI: 0.023, 0.136).

## Discussion

In this study, we have estimated the impact of the COVID-19 pandemic on the service use and well-being of patients with SMI in the 3 months following the first lockdown in England. The results show that patients who were being assessed by clinicians during this time experienced deterioration in their well-being. Overall HoNOS scores (once composition of patients assessed was controlled for) deteriorated, as did the scores of patients being reassessed within a short window of time, bolstering the impression that this was a real deterioration in mean patient well-being. Almost every aspect of mental well-being and functioning was impacted for some group at some point. Longitudinal surveys during this period found that self-reported symptoms for individuals with SMI were broadly stable [10, 18], and that most patients in the UK were satisfied with the support they had received [11]. These measures, along with the documented reduction in aggregate outcomes such as self-harm [7, 8] and suicide [9], may conceal exacerbation in symptoms which could have implications for quality of life and future service use.

The deterioration in patient well-being came at a time when levels of secondary mental health care service use fell. Referrals to community and outpatient services decreased immediately and remained below their pre-COVID-19 trend until the end of the study period (the end of the first lockdown in July 2020). The overall patterns of resource use found in this study confirm those reported elsewhere in the literature. In our study, community and outpatient contacts shifted rapidly to remote provision with no detectable negative impact on contact rates or waiting times, a result reported in other secondary mental healthcare settings [19]. The rate of inpatient admissions was reduced (although not significantly) despite a reduction in contacts with at-home crisis teams. Also, the severity of admissions did not change (as measured by mean length of stay). From this perspective, the changes in the availability of mental health care did not appear to immediately result in greater demand. This is similar to, for example, a study from Cambridgeshire and Peterborough which found an immediate reduction in both supply of and demand for secondary mental health care, followed by a gradual recovery [20].

Our data indicate a difference in the trajectory of assessed outcomes for those with a recorded history of psychosis compared to the wider population of individuals in contact with secondary mental health services. While both groups saw comparable increases in mean total clinician-assessed problems with well-being and social functioning, for individuals with a history of psychosis this change was immediate rather than gradual. Additionally, those with a history of psychosis showed increased problems with nonaccidental self-injury, problem-drinking and drug-taking, and depressed mood not experienced by the wider group of individuals with SMI. Further specific research is needed into the experiences of patients with psychosis during the COVID-19 pandemic.

### Strengths and limitations

The key strength of this study is its comprehensive analysis of the clinical pathway for patients with SMI during the early phase of the COVID-19 pandemic. In analyzing a large number of separate outcomes for patients with SMI across, including by subpopulation, we show a more comprehensive picture of how patients have been impacted by COVID-19 and associated public health measures than other studies have been able to. In particular, the routine use of a comprehensive, clinician-assessed measure of well-being and social functioning (HoNOS) has allowed detailed analysis of patient outcomes during the full-time period of the early months of the pandemic. The simple, linear interrupted time-series design of this study allows a clear, causal understanding of the time-varying impacts of the pandemic across many outcomes while controlling for patient composition and pre-existing trends.

There are however limitations to the interpretations of this study. Firstly, there are limits to the ability of the statistical analyses to control for changes in the composition of patients assessed or admitted during the study period. A lack of formal recording of ICD-10 diagnoses and other patient characteristics mean that some demographic and clinical variation is unaccounted for. Even with improved recording however, the methods cannot completely control for unobservable selection effects in which patients are assessed in each week. It may be the case that the relative likelihood of assessment increased for patients with more severe symptoms following March 2020. Secondly, estimates of the impact of COVID-19 on inpatient admissions and resource use are likely downwardly biased. The configuration of the inpatient wards—alongside the requirement to isolate all new admissions for several days—meant that the number of inpatient beds available was significantly reduced. Any patients sent out of area or to private providers as a result were not captured by these data. Thirdly, the study is restricted to a single NHS Mental Health Trust in South East England during the first 3 months of the pandemic. The OHFT covers only a small, if largely representative, proportion of the secondary mental healthcare services delivered in England, and other stages of the pandemic may have had distinct impacts on services and outcomes. Finally, the estimated impacts on service use cannot completely untangle supply and demand for mental health care. The reduction in referrals to community and outpatient services during lockdown may have been due to a reduction in mental disorders requiring treatment, or it may have been due to the significant difficulty people had in accessing general practice for anything other than COVID-19 at this time.

### Conclusions and implications

Studying the impact of the initial COVID-19 period on patients with SMI has important implications for both the planning of secondary mental health care and future research on the impacts of COVID-19 in the longer term in this patient group. The documented deterioration in mental health in patients already in contact with secondary mental health services at a time when referrals to these services through primary care fell [21] suggests that there may have been some level of unmet need for mental health care during the COVID-19 pandemic. This means that potential opportunities for early interventions and care may have been missed, which may in turn have implications for future levels of need. Future research in this area should consider the distinct impacts of the pandemic on patients with SMI, and the varied experiences of patients receiving secondary mental health care.

## Data Availability

The data were obtained under a Data Sharing Agreement with the Clinical Record Interactive Search (CRIS) System that prohibits using or sharing the data beyond this study.

## Supplementary material

For supplementary material accompanying this paper visit https://doi.org/10.1192/j.eurpsy.2022.22. click here to view supplementary material
