# Supplementary material for: The early impact of the COVID-19 pandemic on patients with severe mental illness: An interrupted time-series study in South-East England
Source: Eur Psychiatry. 2022 May 18;65(1):e31. doi: 10.1192/j.eurpsy.2022.22 (PMC9228594; doi:10.1192/j.eurpsy.2022.22)
Supplement: Supplementary file 1 [file S0924933822000220sup001.docx]

The early impact of the COVID-19 pandemic on patients with Severe Mental Illness: An interrupted time series study in South-East England

# Appendices

## Appendix 1 – Additional results

### Appendix 1.1 – Full results table

| **Outcome** | | **Constant** | **Weekly slope** | **Immediate change** | **Subsequent change** | **Combined change** | **ARIMA terms** |
| --- | --- | --- | --- | --- | --- | --- | --- |
| Outpatient and community referrals: | | Coefficient (95% CI) | Coefficient (95% CI) | Coefficient (95% CI) | Coefficient (95% CI) | Linear combination after 15 weeks (95% CI) | (AR, I, MA) |
|  | Total referrals | 335.2*** (312.3, 358.1) | 0.3*** (0.1, 0.5) | -195.6*** (-300, -91.2) | 17*** (7.4, 26.7) | 60 (-8.3, 128.3) | (1, 0, 0) |
|  | First referrals | 108.8*** (79.1, 138.6) | -0.1 (-0.2, 0.1) | -50.6 (-117.7, 16.5) | 3.3 (-2.9, 9.6) | -0.5 (-41.5, 40.6) | (2, 0, 0) |
|  | Early Intervention in Psychosis | 10.39*** (9.26, 11.52) | -0.01** (-0.02, 0) | -2.11 (-8.22, 4) | 0.26 (-0.38, 0.9) | 1.78 (-3.31, 6.87) | (1, 0, 0) |
|  | Community Mental Health Teams | 37.41** (12.03, 62.79) | 0.12 (-0.06, 0.29) | -54.78*** (-84.64, -24.92) | 5.61*** (3.11, 8.1) | 29.3 (-1.39, 59.98) | (1, 0, 1) |
|  | Crisis Teams | 26.74*** (23.05, 30.43) | 0.04* (0.01, 0.06) | -23.47* (-44.56, -2.39) | 2.29** (0.66, 3.91) | 10.83 (-0.08, 21.74) | (1, 0, 1) |
|  | Psychiatric Liaison | 44.86*** (40, 49.72) | 0.02 (-0.01, 0.06) | -21.2 (-53.26, 10.85) | 1.47 (-1.33, 4.27) | 0.83 (-15.69, 17.34) | (1, 0, 1) |
|  | Mean days to contact after referral | 456.29*** (450.9, 461.69) | -1.67*** (-1.74, -1.6) | -20.04 (-48.99, 8.9) | -1.74 (-5.39, 1.92) | -46.12* (-83.48, -8.77) | (1, 0, 0) |
|  | % referrals with contact within 30 days | 54.6*** (53.2, 55.9) | -0.1*** (-0.1, 0) | 8.1*** (6, 10.3) | 0.4*** (0.2, 0.6) | 14*** (12, 16.1) | (1, 0, 1) |
| Outpatient contacts: | |  |  |  |  |  |  |
|  | Total contacts | 2125.4*** (2028.3, 2222.6) | -0.7* (-1.5, 0) | -26.2 (-475.5, 423.2) | 95.1*** (46.2, 144) | 1400.2*** (997.5, 1802.8) | (0, 0, 1) |
|  | First contacts | 129.4*** (110, 148.7) | -0.1* (-0.3, 0) | -10.4 (-46.8, 26.1) | 0.5 (-2.8, 3.8) | -2.8 (-25.5, 19.9) | (0, 0, 1) |
|  | Face-to-face contacts | 1812*** (1728.7, 1895.2) | -0.9** (-1.5, -0.3) | -1209.3*** (-1695, -723.6) | 16 (-94.8, 126.7) | -969.8 (-2386.3, 446.6) | (0, 0, 1) |
|  | Remote contacts | 271.5*** (119.4, 423.6) | 0.6 (-0.4, 1.6) | 917.3*** (838.1, 996.6) | 85.6*** (69.9, 101.3) | 2201.2*** (1989.3, 2413) | (1, 0, 1) |
|  | Early Intervention in Psychosis | 127.1*** (110.2, 144) | 0.1 (0, 0.3) | 3 (-69.6, 75.6) | 8.4* (0.6, 16.2) | 128.9*** (56.6, 201.2) | (1, 0, 1) |
|  | Community Mental Health Teams | 728.2*** (695.3, 761) | -0.6*** (-0.9, -0.4) | 129.3** (38.3, 220.3) | 19.8*** (9.1, 30.5) | 426.3*** (321.8, 530.8) | (0, 0, 1) |
|  | Crisis Teams | 37.9*** (30.7, 45.1) | 0 (0, 0.1) | -26.1* (-49, -3.2) | 2.2** (0.6, 3.8) | 6.5 (-6.2, 19.2) | (1, 0, 1) |
|  | Psychiatric Liaison | 60.7*** (54.5, 66.9) | 0 (-0.1, 0) | -20.3 (-55.9, 15.3) | 1.5 (-2, 5) | 2 (-23.1, 27.1) | (1, 0, 1) |
| Inpatient admissions: | |  |  |  |  |  |  |
|  | Total admissions | 16.11*** (13.74, 18.49) | -0.01 (-0.03, 0.01) | -4.2 (-9.89, 1.49) | 0.21 (-0.41, 0.83) | -1.03 (-6.85, 4.79) | (1, 0, 1) |
|  | First admissions | 7.02*** (3.92, 10.12) | 0.01 (-0.01, 0.03) | -3.19 (-6.71, 0.34) | 0.14 (-0.23, 0.51) | -1.09 (-4.42, 2.24) | (0, 0, 0) |
|  | Admissions to acute/PICU wards | 10.94*** (9.64, 12.23) | 0 (-0.01, 0.01) | -3.39 (-7.62, 0.84) | 0.19 (-0.29, 0.66) | -0.61 (-5.08, 3.86) | (1, 0, 1) |
|  | % who were discharged in last 30 days | 7.69*** (5.95, 9.42) | -0.01 (-0.03, 0) | -2.58* (-4.97, -0.19) | 0.03 (-0.2, 0.26) | -2.13 (-4.96, 0.7) | (0, 0, 0) |
|  | Total bed days | 1253.1*** (1191.2, 1315) | -1.3*** (-1.9, -0.8) | -1.7 (-54, 50.5) | 0.2 (-12.2, 12.6) | 0.9 (-185.5, 187.3) | (1, 0, 1) |
|  | Unique inpatients | 194.4*** (185, 203.8) | -0.2*** (-0.3, -0.1) | -6.9 (-68.6, 54.8) | 0.3 (-4.1, 4.6) | -2.7 (-29, 23.6) | (1, 0, 0) |
|  | Inpatient length of stay | 90.8*** (83, 98.7) | -0.2*** (-0.3, -0.2) | 3.4 (-7.8, 14.5) | 0.1 (-1, 1.2) | 4.6 (-8.6, 17.9) | (0, 0, 0) |
| HoNOS assessments: | |  |  |  |  |  |  |
|  | Total assessments | 195.93*** (185.78, 206.08) | 0.01 (-0.08, 0.1) | -30.78 (-81.38, 19.81) | 0.89 (-5.2, 6.98) | -17.39 (-69, 34.22) | (0, 0, 1) |
|  | Initial assessments | 97.26*** (91.91, 102.61) | 0.06** (0.02, 0.11) | -21.51 (-50.42, 7.4) | 0.33 (-2.88, 3.53) | -16.62 (-42.37, 9.13) | (0, 0, 1) |
|  | Ongoing assessments | 71.48*** (66.29, 76.66) | -0.05* (-0.09, 0) | -0.39 (-22.89, 22.1) | 0.43 (-2.29, 3.15) | 6.05 (-17.89, 29.98) | (0, 0, 1) |
|  | Discharge assessments | 26.79*** (23.61, 29.97) | 0 (-0.03, 0.03) | -9.7 (-20.52, 1.12) | 0.16 (-1.45, 1.76) | -7.37 (-25.09, 10.35) | (1, 0, 1) |
| HoNOS scores: | |  |  |  |  |  |  |
|  | Mean total score | 12.166*** (12.028, 12.304) | -0.006*** (-0.007, -0.005) | -0.209 (-0.435, 0.017) | 0.06*** (0.033, 0.087) | 0.687*** (0.373, 1.001) | (0, 0, 0) |
| Overactive, aggressive, disruptive or agitated behaviour | |  |  |  |  |  |  |
|  | Any problems | 47.93*** (46.93, 48.92) | -0.01** (-0.02, 0) | 0.42 (-1.21, 2.05) | 0.16 (-0.03, 0.35) | 2.81* (0.55, 5.07) | (0, 0, 0) |
|  | Mean score | 0.896*** (0.872, 0.919) | -0.001*** (-0.001, 0) | -0.09*** (-0.129, -0.052) | 0.015*** (0.01, 0.019) | 0.133*** (0.08, 0.186) | (0, 0, 0) |
| Non-accidental self-injury | |  |  |  |  |  |  |
|  | Any problems | 25.95*** (25.01, 26.9) | 0.03*** (0.02, 0.04) | 1.07 (-0.47, 2.62) | -0.09 (-0.28, 0.09) | -0.34 (-2.48, 1.81) | (0, 0, 0) |
|  | Mean score | 0.517*** (0.496, 0.538) | 0*** (0, 0.001) | -0.04* (-0.074, -0.006) | 0.005* (0.001, 0.009) | 0.032 (-0.015, 0.079) | (0, 0, 0) |
| Problem-drinking or drug-taking | |  |  |  |  |  |  |
|  | Any problems | 23.1*** (22.23, 23.97) | 0.01 (0, 0.01) | -0.95 (-2.37, 0.47) | 0.06 (-0.1, 0.23) | 0.01 (-1.96, 1.98) | (0, 0, 0) |
|  | Mean score | 0.469*** (0.45, 0.488) | 0 (0, 0) | -0.086*** (-0.117, -0.055) | 0.008*** (0.004, 0.011) | 0.027 (-0.016, 0.069) | (0, 0, 0) |
| Cognitive problems | |  |  |  |  |  |  |
|  | Any problems | 46.06*** (44.29, 47.84) | -0.04*** (-0.05, -0.02) | -5.43*** (-6.82, -4.03) | 0.55*** (0.34, 0.76) | 2.85 (-0.06, 5.77) | (2, 0, 1) |
|  | Mean score | 0.944*** (0.907, 0.98) | -0.001*** (-0.001, -0.001) | -0.091*** (-0.125, -0.057) | 0.012*** (0.007, 0.017) | 0.088** (0.027, 0.149) | (1, 0, 1) |
| Physical illness or disability or disability problems | |  |  |  |  |  |  |
|  | Any problems | 50.33*** (48.3, 52.36) | -0.01 (-0.03, 0) | -0.46 (-4.12, 3.2) | 0.03 (-0.26, 0.31) | -0.05 (-2.77, 2.68) | (1, 0, 1) |
|  | Mean score | 1.071*** (1.044, 1.097) | -0.001*** (-0.001, 0) | -0.091** (-0.161, -0.022) | 0.012*** (0.006, 0.018) | 0.084*** (0.045, 0.123) | (2, 0, 0) |
| Problems associated with hallucinations and delusions | |  |  |  |  |  |  |
|  | Any problems | 28.35*** (27.3, 29.41) | 0 (-0.01, 0.01) | 2.89** (0.97, 4.81) | -0.03 (-0.22, 0.16) | 2.48** (0.95, 4.01) | (1, 0, 0) |
|  | Mean score | 0.618*** (0.595, 0.641) | 0** (0, 0) | 0.03 (-0.007, 0.068) | 0.003 (-0.001, 0.008) | 0.08** (0.028, 0.132) | (0, 0, 0) |
| Problems with depressed mood | |  |  |  |  |  |  |
|  | Any problems | 75.64*** (74.83, 76.44) | 0.02*** (0.01, 0.03) | 2.02** (0.7, 3.34) | -0.19* (-0.35, -0.04) | -0.9 (-2.74, 0.93) | (0, 0, 0) |
|  | Mean score | 1.613*** (1.591, 1.636) | 0 (0, 0) | 0.006 (-0.031, 0.043) | -0.001 (-0.005, 0.004) | -0.004 (-0.055, 0.048) | (0, 0, 0) |
| Other mental and behavioural problems | |  |  |  |  |  |  |
|  | Any problems | 77.88*** (77.17, 78.6) | 0.03*** (0.02, 0.04) | 1.87** (0.7, 3.04) | -0.25*** (-0.38, -0.11) | -1.82* (-3.44, -0.19) | (0, 0, 0) |
|  | Mean score | 1.859*** (1.834, 1.885) | 0 (0, 0) | -0.066** (-0.108, -0.024) | 0.007** (0.002, 0.012) | 0.034 (-0.024, 0.092) | (0, 0, 0) |
| Problems with relationships: | |  |  |  |  |  |  |
|  | Any problems | 63.09*** (61.93, 64.24) | -0.02*** (-0.03, -0.01) | 11.16*** (9.27, 13.06) | -0.81*** (-1.04, -0.59) | -1.02 (-3.65, 1.6) | (0, 0, 0) |
|  | Mean score | 1.293*** (1.265, 1.32) | -0.001*** (-0.001, -0.001) | 0.15*** (0.105, 0.195) | -0.014*** (-0.019, -0.009) | -0.058 (-0.121, 0.005) | (0, 0, 0) |
| Problems with activities of daily living | |  |  |  |  |  |  |
|  | Any problems | 61.44*** (60.51, 62.37) | -0.05*** (-0.06, -0.05) | 4.53*** (3.01, 6.05) | -0.07 (-0.25, 0.11) | 3.55** (1.44, 5.66) | (0, 0, 0) |
|  | Mean score | 1.217*** (1.195, 1.239) | -0.001*** (-0.001, -0.001) | -0.017 (-0.052, 0.019) | 0.009*** (0.004, 0.013) | 0.114*** (0.064, 0.163) | (0, 0, 0) |
| Problems with living conditions | |  |  |  |  |  |  |
|  | Any problems | 30.19*** (29.18, 31.19) | -0.02*** (-0.03, -0.02) | 6.4*** (4.76, 8.03) | -0.45*** (-0.64, -0.25) | -0.3 (-2.57, 1.98) | (0, 0, 0) |
|  | Mean score | 0.557*** (0.537, 0.577) | -0.001*** (-0.001, 0) | 0.076*** (0.044, 0.108) | -0.005** (-0.009, -0.001) | -0.001 (-0.045, 0.044) | (0, 0, 0) |
| Problems with occupation and activities | |  |  |  |  |  |  |
|  | Any problems | 58.12*** (57.12, 59.13) | -0.05*** (-0.06, -0.04) | 4.94*** (3.29, 6.58) | 0.02 (-0.17, 0.22) | 5.29*** (3.01, 7.58) | (0, 0, 0) |
|  | Mean score | 1.151*** (1.128, 1.174) | -0.001*** (-0.001, -0.001) | 0.015 (-0.022, 0.053) | 0.007** (0.003, 0.012) | 0.125*** (0.073, 0.177) | (0, 0, 0) |
| HoNOS repeated assessments (within 12 weeks) | |  |  |  |  |  |  |
|  | Mean score change | -1.463*** (-1.739, -1.187) | 0.004** (0.001, 0.006) | -1.727*** (-2.101, -1.353) | 0.352*** (0.322, 0.381) | 3.547*** (3.18, 3.914) | (0, 0, 0) |
|  | % with overall deterioration | 33.08*** (31.42, 34.74) | 0.02*** (0.01, 0.04) | -3.78** (-6.04, -1.53) | 0.67*** (0.49, 0.85) | 6.23*** (4.02, 8.44) | (0, 0, 0) |
|  | % with overall improvement | 55.5*** (53.88, 57.13) | -0.02*** (-0.04, -0.01) | -0.85 (-3.06, 1.35) | -0.23** (-0.41, -0.06) | -4.34*** (-6.49, -2.18) | (0, 0, 0) |

All models estimated with Maximum Likelihood, except where all ARIMA terms are equal to 0 in which case models are estimated with OLS. All models cover the full weeks from March 1^st^, 2016 to July 4^th^, 2020 except for first admissions/referrals, which start from March 1^st^, 2018.
Significance levels: **p<0.1, **p<0.05,*  ****p<0.01*

### Appendix 1.2 – Results excluding January to March 2020

| **Outcome** | | **Constant** | **Weekly slope** | **Step change** | **Slope change** | **Combined (15 weeks)** | **ARIMA terms** |
| --- | --- | --- | --- | --- | --- | --- | --- |
| Outpatient referrals: | | Coefficient (95% CI) | Coefficient (95% CI) | Coefficient (95% CI) | Coefficient (95% CI) | Linear combination after 15 weeks (95% CI) | (AR, I, MA) |
|  | Total referrals | 344.8*** (325.1, 364.4) | 0.2* (0, 0.3) | -174.9*** (-260.4, -89.4) | 16.9*** (9, 24.9) | 79.2** (19.4, 138.9) | (1, 0, 0) |
|  | First referrals | 131.7*** (106.4, 156.9) | -0.2** (-0.4, -0.1) | -35.1 (-74.3, 4.1) | 3.2 (-0.8, 7.2) | 13.2 (-18.7, 45.1) | (2, 0, 0) |
|  | Early Intervention in Psychosis | 10.51*** (9.35, 11.67) | -0.01** (-0.02, 0) | -2.08 (-7.96, 3.8) | 0.28 (-0.34, 0.9) | 2.12 (-2.98, 7.23) | (1, 0, 0) |
|  | Community Mental Health Teams | 47.83*** (44.68, 50.98) | -0.03* (-0.06, 0) | 5.38 (-2.51, 13.27) | 3.69*** (2.85, 4.53) | 60.74*** (52.22, 69.26) | (1, 0, 1) |
|  | Crisis Teams | 28.77*** (26.66, 30.89) | 0.01 (-0.01, 0.03) | -14.58* (-25.74, -3.41) | 1.95*** (0.96, 2.94) | 14.64*** (7.34, 21.95) | (1, 0, 1) |
|  | Psychiatric Liaison | 44.05*** (39.31, 48.78) | 0.03 (0, 0.07) | -30.22* (-55.52, -4.91) | 2 (-0.24, 4.24) | -0.16 (-14.9, 14.57) | (1, 0, 1) |
|  | Mean days to contact after referral | 456.01*** (450.32, 461.71) | -1.67*** (-1.74, -1.59) | -20.45 (-51.46, 10.55) | -1.76 (-5.47, 1.95) | -46.8* (-84.39, -9.2) | (1, 0, 0) |
|  | % referrals with contact within 30 days | 54.1*** (53.1, 55.1) | 0*** (-0.1, 0) | 5.3*** (3.7, 6.8) | 0.6*** (0.4, 0.7) | 13.6*** (11.9, 15.2) | (1, 0, 1) |
| Outpatient contacts: | |  |  |  |  |  |  |
|  | Total contacts | 2135.5*** (2034.8, 2236.1) | -0.9* (-1.7, -0.1) | -18.5 (-456.5, 419.6) | 96.5*** (49, 144) | 1429*** (1025.2, 1832.8) | (0, 0, 1) |
|  | First contacts | 133.6*** (111.8, 155.3) | -0.2* (-0.3, 0) | -9 (-51, 33) | 0.6 (-3.1, 4.4) | 0.3 (-24.8, 25.4) | (0, 0, 1) |
|  | Face-to-face contacts | 1814.9*** (1731.6, 1898.3) | -1** (-1.6, -0.3) | -1274.6 (-2734.8, 185.6) | 22.8 (-164.4, 209.9) | -933.1 (-2809.3, 943.1) | (0, 0, 1) |
|  | Remote contacts | 296.7*** (211.4, 382) | 0.2 (-0.5, 0.9) | 1201.1*** (1103.3, 1299) | 73.9*** (67, 80.8) | 2310.2*** (2196.2, 2424.1) | (1, 0, 1) |
|  | Early Intervention in Psychosis | 128.6*** (110.5, 146.8) | 0.1 (0, 0.3) | 6.2 (-98, 110.4) | 8.4 (-1.5, 18.3) | 131.8*** (53.8, 209.9) | (1, 0, 1) |
|  | Community Mental Health Teams | 731.7*** (697.5, 765.9) | -0.7*** (-0.9, -0.4) | 131.6** (39.5, 223.7) | 20.3*** (9.8, 30.9) | 436.6*** (331.2, 541.9) | (0, 0, 1) |
|  | Crisis Teams | 38.5*** (31.9, 45.1) | 0 (-0.1, 0.1) | -25.5 (-54.6, 3.7) | 2.2* (0.2, 4.2) | 7 (-5.8, 19.9) | (1, 0, 1) |
|  | Psychiatric Liaison | 59.4*** (53.5, 65.3) | 0 (-0.1, 0) | -35.8** (-57.4, -14.2) | 2.5* (0.3, 4.8) | 2.2 (-17.9, 22.3) | (1, 0, 1) |
| Inpatient admissions: | |  |  |  |  |  |  |
|  | Total admissions | 16.31*** (14, 18.62) | -0.01 (-0.03, 0.01) | -3.36 (-9.85, 3.12) | 0.2 (-0.44, 0.84) | -0.37 (-6.33, 5.59) | (1, 0, 1) |
|  | First admissions | 7.8*** (4.29, 11.31) | 0.01 (-0.01, 0.03) | -2.79 (-6.42, 0.84) | 0.15 (-0.22, 0.51) | -0.61 (-4.1, 2.88) | (0, 0, 0) |
|  | Admissions to acute/PICU wards | 10.99*** (9.59, 12.38) | 0 (-0.02, 0.01) | -3.09 (-7.57, 1.4) | 0.17 (-0.3, 0.64) | -0.56 (-5.02, 3.9) | (1, 0, 1) |
|  | % who were discharged in last 30 days | 7.46*** (5.67, 9.25) | -0.01 (-0.02, 0.01) | -2.92* (-5.46, -0.38) | 0.02 (-0.21, 0.25) | -2.59 (-5.57, 0.38) | (0, 0, 0) |
|  | Total bed days | 1252.9*** (1191, 1314.7) | -1.3*** (-1.9, -0.7) | -28.4 (-847.5, 790.6) | 1.6 (-49.7, 52.9) | -5 (-194.7, 184.6) | (1, 0, 1) |
|  | Unique inpatients | 194.6*** (185.1, 204.1) | -0.2*** (-0.3, -0.1) | -8.3 (-71.7, 55.1) | 0.4 (-4, 4.8) | -2.5 (-29.3, 24.3) | (1, 0, 0) |
|  | Inpatient length of stay | 90.8*** (82.7, 99) | -0.2*** (-0.3, -0.2) | 3.7 (-8.2, 15.6) | 0.1 (-1, 1.2) | 4.7 (-9.3, 18.7) | (0, 0, 0) |
| HoNOS assessments: | |  |  |  |  |  |  |
|  | Total assessments | 196.59*** (186.03, 207.14) | 0 (-0.09, 0.1) | -32.31 (-79.31, 14.68) | 1.17 (-4.51, 6.86) | -14.71 (-65.27, 35.84) | (0, 0, 1) |
|  | Initial assessments | 98.07*** (92.53, 103.61) | 0.05* (0, 0.1) | -20.46 (-48.17, 7.24) | 0.39 (-2.71, 3.5) | -14.57 (-40.44, 11.31) | (0, 0, 1) |
|  | Ongoing assessments | 71.28*** (65.89, 76.68) | -0.04 (-0.09, 0) | -2.49 (-24.39, 19.41) | 0.59 (-2.02, 3.2) | 6.37 (-17.18, 29.91) | (0, 0, 1) |
|  | Discharge assessments | 27*** (23.66, 30.35) | 0 (-0.03, 0.03) | -7.58 (-20.3, 5.15) | 0.03 (-1.7, 1.76) | -7.18 (-25.52, 11.17) | (1, 0, 1) |
| HoNOS scores: | |  |  |  |  |  |  |
|  | Mean total score | 12.136*** (11.995, 12.278) | -0.005*** (-0.007, -0.004) | -0.27* (-0.504, -0.036) | 0.057*** (0.03, 0.084) | 0.581*** (0.259, 0.903) | (0, 0, 0) |
| Overactive, aggressive, disruptive or agitated behaviour | |  |  |  |  |  |  |
|  | Any problems | 47.8*** (46.76, 48.84) | -0.01* (-0.02, 0) | 0.17 (-1.56, 1.89) | 0.17 (-0.03, 0.36) | 2.66* (0.28, 5.03) | (0, 0, 0) |
|  | Mean score | 0.893*** (0.868, 0.917) | -0.001*** (-0.001, 0) | -0.096*** (-0.137, -0.056) | 0.015*** (0.01, 0.019) | 0.125*** (0.069, 0.18) | (0, 0, 0) |
| Non-accidental self-injury | |  |  |  |  |  |  |
|  | Any problems | 25.87*** (24.89, 26.85) | 0.03*** (0.02, 0.04) | 0.89 (-0.73, 2.51) | -0.1 (-0.29, 0.09) | -0.6 (-2.84, 1.63) | (0, 0, 0) |
|  | Mean score | 0.521*** (0.5, 0.542) | 0*** (0, 0.001) | -0.033 (-0.068, 0.002) | 0.005* (0.001, 0.009) | 0.041 (-0.008, 0.089) | (0, 0, 0) |
| Problem-drinking or drug-taking | |  |  |  |  |  |  |
|  | Any problems | 22.95*** (22.07, 23.82) | 0.01 (0, 0.01) | -1.27 (-2.71, 0.18) | 0.04 (-0.13, 0.2) | -0.74 (-2.72, 1.25) | (0, 0, 0) |
|  | Mean score | 0.466*** (0.447, 0.486) | 0 (0, 0) | -0.092*** (-0.124, -0.059) | 0.007*** (0.003, 0.011) | 0.016 (-0.028, 0.061) | (0, 0, 0) |
| Cognitive problems | |  |  |  |  |  |  |
|  | Any problems | 46.47*** (44.8, 48.13) | -0.04*** (-0.06, -0.03) | -3.18* (-5.65, -0.71) | 0.49*** (0.3, 0.68) | 4.16** (1.34, 6.99) | (2, 0, 1) |
|  | Mean score | 0.949*** (0.912, 0.985) | -0.001*** (-0.001, -0.001) | -0.055 (-0.114, 0.004) | 0.011*** (0.006, 0.015) | 0.102** (0.04, 0.165) | (1, 0, 1) |
| Physical illness or disability or disability problems | |  |  |  |  |  |  |
|  | Any problems | 50.21*** (48.53, 51.89) | -0.01 (-0.03, 0.01) | -1.37 (-5.19, 2.45) | 0.02 (-0.25, 0.29) | -1.07 (-4.05, 1.92) | (1, 0, 1) |
|  | Mean score | 1.066*** (1.04, 1.091) | 0*** (-0.001, 0) | -0.114*** (-0.178, -0.051) | 0.012*** (0.007, 0.017) | 0.064** (0.025, 0.104) | (2, 0, 0) |
| Problems associated with hallucinations and delusions | |  |  |  |  |  |  |
|  | Any problems | 28.04*** (27.01, 29.08) | 0 (0, 0.01) | 2.18* (0.3, 4.06) | -0.02 (-0.21, 0.16) | 1.83* (0.31, 3.36) | (1, 0, 0) |
|  | Mean score | 0.612*** (0.588, 0.636) | 0 (0, 0) | 0.018 (-0.021, 0.057) | 0.003 (-0.001, 0.008) | 0.066* (0.012, 0.12) | (0, 0, 0) |
| Problems with depressed mood | |  |  |  |  |  |  |
|  | Any problems | 75.62*** (74.79, 76.44) | 0.02*** (0.01, 0.03) | 1.98** (0.61, 3.35) | -0.19* (-0.34, -0.03) | -0.83 (-2.71, 1.05) | (0, 0, 0) |
|  | Mean score | 1.612*** (1.589, 1.636) | 0 (0, 0) | 0.004 (-0.035, 0.042) | -0.001 (-0.005, 0.004) | -0.007 (-0.06, 0.046) | (0, 0, 0) |
| Other mental and behavioural problems | |  |  |  |  |  |  |
|  | Any problems | 77.75*** (77.02, 78.47) | 0.03*** (0.03, 0.04) | 1.61** (0.4, 2.81) | -0.26*** (-0.4, -0.12) | -2.28** (-3.94, -0.62) | (0, 0, 0) |
|  | Mean score | 1.858*** (1.832, 1.885) | 0 (0, 0) | -0.068** (-0.111, -0.024) | 0.006* (0.001, 0.011) | 0.029 (-0.032, 0.089) | (0, 0, 0) |
| Problems with relationships: | |  |  |  |  |  |  |
|  | Any problems | 63*** (61.81, 64.2) | -0.02** (-0.03, -0.01) | 11*** (9.02, 12.98) | -0.8*** (-1.03, -0.58) | -1.04 (-3.77, 1.69) | (0, 0, 0) |
|  | Mean score | 1.29*** (1.262, 1.319) | -0.001*** (-0.001, -0.001) | 0.145*** (0.098, 0.193) | -0.014*** (-0.019, -0.008) | -0.062 (-0.127, 0.003) | (0, 0, 0) |
| Problems with activities of daily living | |  |  |  |  |  |  |
|  | Any problems | 61.36*** (60.42, 62.31) | -0.05*** (-0.06, -0.05) | 4.37*** (2.8, 5.94) | -0.09 (-0.27, 0.09) | 3.01** (0.86, 5.17) | (0, 0, 0) |
|  | Mean score | 1.212*** (1.19, 1.234) | -0.001*** (-0.001, -0.001) | -0.026 (-0.063, 0.01) | 0.008*** (0.004, 0.012) | 0.096*** (0.046, 0.146) | (0, 0, 0) |
| Problems with living conditions | |  |  |  |  |  |  |
|  | Any problems | 30.08*** (29.03, 31.13) | -0.02*** (-0.03, -0.01) | 6.19*** (4.46, 7.93) | -0.45*** (-0.65, -0.25) | -0.54 (-2.92, 1.85) | (0, 0, 0) |
|  | Mean score | 0.555*** (0.534, 0.575) | -0.001*** (-0.001, 0) | 0.071*** (0.038, 0.105) | -0.005** (-0.009, -0.002) | -0.01 (-0.056, 0.037) | (0, 0, 0) |
| Problems with occupation and activities | |  |  |  |  |  |  |
|  | Any problems | 57.88*** (56.89, 58.87) | -0.04*** (-0.05, -0.04) | 4.45*** (2.81, 6.08) | -0.02 (-0.21, 0.17) | 4.18*** (1.93, 6.44) | (0, 0, 0) |
|  | Mean score | 1.146*** (1.123, 1.169) | -0.001*** (-0.001, -0.001) | 0.006 (-0.032, 0.043) | 0.007** (0.002, 0.011) | 0.104*** (0.052, 0.156) | (0, 0, 0) |
| HoNOS repeated assessments (within 12 weeks) | |  |  |  |  |  |  |
|  | Mean score change | -1.35*** (-1.629, -1.07) | 0.002 (0, 0.004) | -1.509*** (-1.9, -1.118) | 0.353*** (0.324, 0.382) | 3.786*** (3.397, 4.174) | (0, 0, 0) |
|  | % with overall deterioration | 33.54*** (31.87, 35.21) | 0.02** (0, 0.03) | -3.03* (-5.36, -0.69) | 0.67*** (0.5, 0.85) | 7.04*** (4.72, 9.36) | (0, 0, 0) |
|  | % with overall improvement | 54.94*** (53.3, 56.58) | -0.02* (-0.03, 0) | -1.93 (-4.22, 0.37) | -0.24** (-0.41, -0.07) | -5.51*** (-7.79, -3.23) | (0, 0, 0) |

All models estimated with Maximum Likelihood, except where all ARIMA terms are equal to 0 in which case models are estimated with OLS. All models cover the full weeks from March 1^st^, 2016 to July 4^th^, 2020 except for first admissions/referrals, which start from March 1^st^, 2018.
Significance levels: **p<0.1, **p<0.05,*  ****p<0.01*

### Appendix 1.3 – Results for patients from deprived areas

| **Outcome** | | **Constant** | **Weekly slope** | **Step change** | **Slope change** | **Combined (15 weeks)** | **ARIMA terms** |
| --- | --- | --- | --- | --- | --- | --- | --- |
| Outpatient referrals: | | Coefficient (95% CI) | Coefficient (95% CI) | Coefficient (95% CI) | Coefficient (95% CI) | Linear combination after 15 weeks (95% CI) | (AR, I, MA) |
|  | Total referrals | 39.4*** (36.8, 41.9) | 0 (0, 0) | -16.8** (-28.9, -4.8) | 1.9*** (0.8, 3.1) | 12.1* (2.7, 21.6) | (0, 0, 1) |
|  | Mean days to contact after referral | 464.95*** (452.54, 477.36) | -1.69*** (-1.79, -1.59) | -31.37*** (-48.34, -14.4) | -0.78 (-1.85, 0.3) | -43.03*** (-58.31, -27.76) | (0, 0, 0) |
|  | % referrals with contact within 30 days | 56.4*** (54.5, 58.3) | -0.1*** (-0.1, -0.1) | 8.2*** (5.6, 10.8) | 0.1 (0, 0.3) | 10.3*** (8, 12.6) | (0, 0, 0) |
| Outpatient contacts: | |  |  |  |  |  |  |
|  | Total contacts | 212.9*** (202.2, 223.6) | -0.2*** (-0.3, -0.1) | -15.8 (-87.4, 55.7) | 11.1** (4.1, 18.1) | 150.9*** (101.4, 200.3) | (1, 0, 0) |
|  | Face-to-face contacts | 171.9*** (163.4, 180.3) | -0.2*** (-0.2, -0.1) | -98.6 (-204.3, 7.1) | 2.3 (-6.4, 11.1) | -63.5* (-118.6, -8.3) | (1, 0, 0) |
|  | Remote contacts | 39.1*** (33.4, 44.9) | 0 (-0.1, 0) | 79.9*** (68.2, 91.7) | 8.7*** (7.5, 10) | 210.6*** (197.8, 223.3) | (1, 0, 1) |
| Inpatient admissions: | |  |  |  |  |  |  |
|  | Total bed days | 190.3*** (164.7, 215.9) | -0.1 (-0.3, 0.1) | -7.1 (-43.6, 29.4) | -0.8 (-6.5, 4.9) | -18.8 (-100.7, 63.1) | (2, 0, 0) |
| HoNOS assessments: | |  |  |  |  |  |  |
|  | Total assessments | 15.86*** (14.57, 17.15) | 0 (-0.01, 0.01) | -4.92 (-10.12, 0.28) | 0.51 (-0.05, 1.06) | 2.72 (-2.06, 7.5) | (0, 0, 0) |
| HoNOS scores: | |  |  |  |  |  |  |
|  | Mean total score | 12.992*** (12.506, 13.477) | -0.004* (-0.008, -0.001) | -2.504*** (-3.217, -1.79) | 0.179*** (0.122, 0.237) | 0.188 (-0.497, 0.873) | (0, 0, 0) |
| Overactive, aggressive, disruptive or agitated behaviour | |  |  |  |  |  |  |
|  | Any problems | 56.92*** (52.85, 60.99) | -0.03 (-0.06, 0) | -5.38 (-11.35, 0.6) | 0.67** (0.19, 1.15) | 4.66 (-1.07, 10.4) | (0, 0, 0) |
|  | Mean score | 1.106*** (1.023, 1.189) | -0.001*** (-0.002, -0.001) | -0.095 (-0.217, 0.027) | 0.016** (0.006, 0.026) | 0.147* (0.03, 0.264) | (0, 0, 0) |
| Non-accidental self-injury | |  |  |  |  |  |  |
|  | Any problems | 31*** (27.6, 34.4) | 0.04** (0.01, 0.06) | -0.24 (-5.24, 4.76) | -0.67** (-1.07, -0.27) | -10.31*** (-15.11, -5.52) | (0, 0, 0) |
|  | Mean score | 0.666*** (0.6, 0.732) | 0 (0, 0.001) | -0.133** (-0.229, -0.036) | -0.006 (-0.014, 0.002) | -0.222*** (-0.315, -0.13) | (0, 0, 0) |
| Problem-drinking or drug-taking | |  |  |  |  |  |  |
|  | Any problems | 34.06*** (30.56, 37.55) | 0.01 (-0.02, 0.03) | 1.89 (-3.25, 7.03) | 0.06 (-0.35, 0.48) | 2.83 (-2.09, 7.76) | (0, 0, 0) |
|  | Mean score | 0.771*** (0.689, 0.854) | 0 (-0.001, 0) | -0.029 (-0.15, 0.092) | 0.01* (0, 0.02) | 0.12* (0.004, 0.236) | (0, 0, 0) |
| Cognitive problems | |  |  |  |  |  |  |
|  | Any problems | 34.3*** (31.08, 37.52) | 0 (-0.03, 0.02) | -3.3 (-8.03, 1.43) | 0.71*** (0.33, 1.09) | 7.41** (2.87, 11.95) | (0, 0, 0) |
|  | Mean score | 0.626*** (0.551, 0.701) | 0 (-0.001, 0) | -0.06 (-0.17, 0.051) | 0.012* (0.003, 0.02) | 0.113* (0.007, 0.219) | (0, 0, 0) |
| Physical illness or disability or disability problems | |  |  |  |  |  |  |
|  | Any problems | 42.77*** (40.4, 45.14) | 0 (-0.02, 0.02) | 1.97 (-0.99, 4.92) | -0.64*** (-0.88, -0.4) | -7.58*** (-10.94, -4.21) | (3, 0, 0) |
|  | Mean score | 0.91*** (0.852, 0.968) | 0 (-0.001, 0) | -0.123** (-0.199, -0.046) | 0.003 (-0.002, 0.009) | -0.073* (-0.143, -0.002) | (3, 0, 0) |
| Problems associated with hallucinations and delusions | |  |  |  |  |  |  |
|  | Any problems | 32.33*** (29.45, 35.22) | 0.01 (-0.01, 0.03) | -14.8*** (-19.04, -10.56) | 1.29*** (0.95, 1.63) | 4.55* (0.48, 8.62) | (0, 0, 0) |
|  | Mean score | 0.702*** (0.623, 0.781) | 0 (-0.001, 0) | -0.364*** (-0.48, -0.248) | 0.034*** (0.025, 0.044) | 0.15** (0.038, 0.261) | (0, 0, 0) |
| Problems with depressed mood | |  |  |  |  |  |  |
|  | Any problems | 81.42*** (79.1, 83.74) | 0.02* (0, 0.04) | 2.1 (-1.31, 5.5) | -0.65*** (-0.92, -0.37) | -7.58*** (-10.85, -4.31) | (0, 0, 0) |
|  | Mean score | 1.697*** (1.617, 1.777) | 0 (-0.001, 0.001) | -0.118* (-0.236, 0) | -0.001 (-0.01, 0.009) | -0.131* (-0.244, -0.018) | (0, 0, 0) |
| Other mental and behavioural problems | |  |  |  |  |  |  |
|  | Any problems | 83.8*** (81.13, 86.48) | 0.03** (0.01, 0.05) | -9.77*** (-13.7, -5.84) | 0.93*** (0.61, 1.24) | 4.13* (0.36, 7.89) | (0, 0, 0) |
|  | Mean score | 1.955*** (1.873, 2.038) | 0 (0, 0.001) | -0.375*** (-0.496, -0.254) | 0.029*** (0.019, 0.039) | 0.061 (-0.055, 0.177) | (0, 0, 0) |
| Problems with relationships: | |  |  |  |  |  |  |
|  | Any problems | 76.78*** (73.77, 79.79) | -0.03* (-0.05, -0.01) | 5.51* (1.08, 9.93) | -0.5** (-0.86, -0.15) | -2.02 (-6.27, 2.22) | (0, 0, 0) |
|  | Mean score | 1.584*** (1.491, 1.677) | -0.001** (-0.002, 0) | -0.126 (-0.263, 0.011) | -0.004 (-0.015, 0.007) | -0.19** (-0.322, -0.059) | (0, 0, 0) |
| Problems with activities of daily living | |  |  |  |  |  |  |
|  | Any problems | 62.42*** (58.91, 65.93) | -0.04* (-0.06, -0.01) | -6.59* (-11.75, -1.44) | 0.35 (-0.07, 0.76) | -1.36 (-6.31, 3.59) | (0, 0, 0) |
|  | Mean score | 1.169*** (1.079, 1.259) | -0.001* (-0.002, 0) | -0.294*** (-0.425, -0.162) | 0.019*** (0.008, 0.03) | -0.008 (-0.135, 0.118) | (0, 0, 0) |
| Problems with living conditions | |  |  |  |  |  |  |
|  | Any problems | 36.4*** (32.91, 39.9) | -0.01 (-0.03, 0.02) | -26.52*** (-31.65, -21.38) | 2.23*** (1.82, 2.64) | 6.91** (1.98, 11.83) | (0, 0, 0) |
|  | Mean score | 0.704*** (0.613, 0.795) | 0 (-0.001, 0) | -0.533*** (-0.667, -0.399) | 0.048*** (0.037, 0.059) | 0.187** (0.058, 0.315) | (0, 0, 0) |
| Problems with occupation and activities | |  |  |  |  |  |  |
|  | Any problems | 60.62*** (57.43, 63.81) | -0.02 (-0.04, 0.01) | -3.91 (-8.6, 0.77) | 0.27 (-0.1, 0.65) | 0.19 (-4.31, 4.68) | (0, 0, 0) |
|  | Mean score | 1.16*** (1.059, 1.262) | -0.001 (-0.001, 0) | -0.32*** (-0.469, -0.171) | 0.024*** (0.012, 0.036) | 0.038 (-0.105, 0.181) | (0, 0, 0) |

All models estimated with Maximum Likelihood, except where all ARIMA terms are equal to 0 in which case models are estimated with OLS. All models cover the full weeks from March 1^st^, 2016 to July 4^th^, 2020 except for first admissions/referrals, which start from March 1^st^, 2018.
Significance levels: **p<0.1, **p<0.05,*  ****p<0.01*

### Appendix 1.4 – Results for patients with a recorded diagnosis of psychosis

| **Outcome** | | **Constant** | **Weekly slope** | **Step change** | **Slope change** | **Combined (15 weeks)** | **ARIMA terms** |
| --- | --- | --- | --- | --- | --- | --- | --- |
| Outpatient referrals: | | Coefficient (95% CI) | Coefficient (95% CI) | Coefficient (95% CI) | Coefficient (95% CI) | Linear combination after 15 weeks (95% CI) | (AR, I, MA) |
|  | Total referrals | 51.9*** (49.4, 54.5) | 0*** (-0.1, 0) | -10.8 (-25, 3.4) | 1 (-0.9, 2.8) | 3.6 (-14.4, 21.6) | (1, 0, 0) |
|  | Mean days to contact after referral | 307.53*** (299.74, 315.31) | -1.01*** (-1.07, -0.95) | -16.91*** (-25.54, -8.28) | -2.35*** (-3.49, -1.21) | -52.1*** (-68.5, -35.7) | (0, 0, 0) |
|  | % referrals with contact within 30 days | 64.6*** (63.3, 65.9) | 0*** (-0.1, 0) | -7.9*** (-9.3, -6.4) | 1.5*** (1.3, 1.6) | 14*** (11.3, 16.7) | (0, 0, 0) |
| Outpatient contacts: | |  |  |  |  |  |  |
|  | Total contacts | 740.4*** (712, 768.8) | -0.2 (-0.4, 0) | 48.2 (-99.9, 196.3) | 26** (10.5, 41.5) | 438.2*** (314.2, 562.2) | (0, 0, 1) |
|  | Face-to-face contacts | 671*** (646.1, 695.9) | -0.4*** (-0.6, -0.2) | -362.8** (-600.7, -124.9) | 8.4 (-22.2, 38.9) | -237.1 (-549.6, 75.5) | (0, 0, 1) |
|  | Remote contacts | 60.4*** (45.1, 75.8) | 0.3*** (0.2, 0.4) | 397.6*** (378.7, 416.6) | 18.4*** (16.4, 20.4) | 673.6*** (655.6, 691.6) | (0, 0, 1) |
| Inpatient admissions: | |  |  |  |  |  |  |
|  | Total bed days | 890.9*** (851.8, 930.1) | -0.9*** (-1.2, -0.5) | 24.8 (-118.2, 167.7) | -1.7 (-14, 10.7) | -0.1 (-135.9, 135.6) | (1, 0, 1) |
| HoNOS assessments: | |  |  |  |  |  |  |
|  | Total assessments | 44.17*** (40.97, 47.36) | 0 (-0.03, 0.02) | 0.19 (-12.3, 12.68) | 0.05 (-1.35, 1.44) | 0.89 (-10.94, 12.72) | (0, 0, 1) |
| HoNOS scores: | |  |  |  |  |  |  |
|  | Mean total score | 13.055*** (12.768, 13.342) | -0.009*** (-0.012, -0.007) | 0.63*** (0.261, 0.999) | 0.008 (-0.02, 0.037) | 0.757*** (0.359, 1.154) | (0, 0, 0) |
| Overactive, aggressive, disruptive or agitated behaviour | |  |  |  |  |  |  |
|  | Any problems | 49.57*** (47.71, 51.43) | -0.04*** (-0.05, -0.03) | 4.43*** (2.04, 6.82) | -0.35*** (-0.54, -0.17) | -0.88 (-3.46, 1.69) | (0, 0, 0) |
|  | Mean score | 0.928*** (0.88, 0.976) | -0.001*** (-0.001, -0.001) | -0.002 (-0.063, 0.059) | 0.004 (-0.001, 0.009) | 0.061 (-0.005, 0.128) | (0, 0, 0) |
| Non-accidental self-injury | |  |  |  |  |  |  |
|  | Any problems | 19.5*** (17.8, 21.21) | -0.02*** (-0.04, -0.01) | 2.91** (0.71, 5.1) | 0.32*** (0.15, 0.49) | 7.75*** (5.38, 10.11) | (0, 0, 0) |
|  | Mean score | 0.37*** (0.335, 0.406) | -0.001*** (-0.001, 0) | -0.018 (-0.064, 0.027) | 0.014*** (0.011, 0.018) | 0.198*** (0.149, 0.247) | (0, 0, 0) |
| Problem-drinking or drug-taking | |  |  |  |  |  |  |
|  | Any problems | 24.88*** (23.21, 26.55) | -0.01 (-0.02, 0.01) | -4.28*** (-6.42, -2.13) | 0.77*** (0.6, 0.93) | 7.2*** (4.89, 9.52) | (0, 0, 0) |
|  | Mean score | 0.512*** (0.474, 0.551) | 0 (0, 0) | -0.13*** (-0.18, -0.08) | 0.02*** (0.016, 0.024) | 0.172*** (0.118, 0.226) | (0, 0, 0) |
| Cognitive problems | |  |  |  |  |  |  |
|  | Any problems | 45.17*** (43.05, 47.29) | -0.02* (-0.03, 0) | -3.25* (-5.97, -0.52) | 0.62*** (0.41, 0.83) | 6.03*** (3.09, 8.96) | (0, 0, 0) |
|  | Mean score | 0.769*** (0.722, 0.815) | 0 (-0.001, 0) | -0.099** (-0.159, -0.039) | 0.017*** (0.013, 0.022) | 0.16*** (0.095, 0.224) | (0, 0, 0) |
| Physical illness or disability or disability problems | |  |  |  |  |  |  |
|  | Any problems | 51.82*** (49.41, 54.22) | 0 (-0.02, 0.01) | 6.95*** (3.87, 10.04) | -0.54*** (-0.78, -0.3) | -1.16 (-4.49, 2.17) | (0, 0, 0) |
|  | Mean score | 1.027*** (0.979, 1.076) | 0 (-0.001, 0) | 0.06* (0.01, 0.109) | 0.008*** (0.005, 0.01) | 0.172*** (0.12, 0.224) | (1, 0, 0) |
| Problems associated with hallucinations and delusions | |  |  |  |  |  |  |
|  | Any problems | 67.68*** (66.47, 68.9) | -0.03*** (-0.04, -0.03) | 3.4*** (1.99, 4.81) | -0.18** (-0.31, -0.04) | 0.77 (-0.6, 2.13) | (4, 0, 2) |
|  | Mean score | 1.591*** (1.535, 1.647) | -0.001*** (-0.002, -0.001) | 0.052 (-0.019, 0.124) | -0.003 (-0.009, 0.002) | 0.001 (-0.076, 0.078) | (0, 0, 0) |
| Problems with depressed mood | |  |  |  |  |  |  |
|  | Any problems | 72.53*** (70.72, 74.34) | -0.02** (-0.04, -0.01) | 5.25*** (2.92, 7.57) | -0.38*** (-0.56, -0.2) | -0.45 (-2.96, 2.05) | (0, 0, 0) |
|  | Mean score | 1.446*** (1.405, 1.487) | -0.001*** (-0.002, -0.001) | 0.145*** (0.093, 0.198) | -0.004* (-0.009, 0) | 0.079** (0.023, 0.136) | (0, 0, 0) |
| Other mental and behavioural problems | |  |  |  |  |  |  |
|  | Any problems | 77.86*** (75.94, 79.79) | 0 (-0.01, 0.02) | -2.13 (-4.61, 0.35) | -0.17 (-0.37, 0.02) | -4.7*** (-7.37, -2.03) | (0, 0, 0) |
|  | Mean score | 1.772*** (1.724, 1.821) | -0.001** (-0.001, 0) | -0.068* (-0.13, -0.005) | 0.004 (-0.001, 0.009) | -0.01 (-0.078, 0.057) | (0, 0, 0) |
| Problems with relationships: | |  |  |  |  |  |  |
|  | Any problems | 67.11*** (65.35, 68.88) | -0.02* (-0.03, 0) | 2.65* (0.38, 4.92) | -0.71*** (-0.89, -0.53) | -8.01*** (-10.45, -5.56) | (0, 0, 0) |
|  | Mean score | 1.383*** (1.338, 1.429) | -0.001*** (-0.002, -0.001) | 0.172*** (0.114, 0.23) | -0.021*** (-0.025, -0.016) | -0.138*** (-0.201, -0.075) | (0, 0, 0) |
| Problems with activities of daily living | |  |  |  |  |  |  |
|  | Any problems | 67.22*** (64.99, 69.45) | -0.05*** (-0.06, -0.03) | 13.09*** (10.22, 15.95) | -1.06*** (-1.29, -0.84) | -2.86 (-5.95, 0.23) | (0, 0, 0) |
|  | Mean score | 1.318*** (1.26, 1.375) | -0.001*** (-0.002, -0.001) | 0.145*** (0.071, 0.219) | -0.011*** (-0.017, -0.005) | -0.02 (-0.099, 0.06) | (0, 0, 0) |
| Problems with living conditions | |  |  |  |  |  |  |
|  | Any problems | 37.21*** (35.33, 39.09) | -0.01 (-0.03, 0) | 5.42*** (3.01, 7.84) | -0.49*** (-0.68, -0.3) | -1.96 (-4.56, 0.65) | (0, 0, 0) |
|  | Mean score | 0.685*** (0.649, 0.722) | 0** (-0.001, 0) | 0.12*** (0.073, 0.167) | -0.012*** (-0.015, -0.008) | -0.054* (-0.105, -0.003) | (0, 0, 0) |
| Problems with occupation and activities | |  |  |  |  |  |  |
|  | Any problems | 63.11*** (61.11, 65.12) | -0.02** (-0.04, -0.01) | 13.22*** (10.65, 15.8) | -0.54*** (-0.75, -0.34) | 5.07*** (2.29, 7.85) | (0, 0, 0) |
|  | Mean score | 1.291*** (1.242, 1.34) | -0.001*** (-0.001, -0.001) | 0.219*** (0.156, 0.282) | -0.007** (-0.012, -0.002) | 0.119*** (0.051, 0.186) | (0, 0, 0) |

All models estimated with Maximum Likelihood, except where all ARIMA terms are equal to 0 in which case models are estimated with OLS. All models cover the full weeks from March 1^st^, 2016 to July 4^th^, 2020 except for first admissions/referrals, which start from March 1^st^, 2018.
Significance levels: **p<0.1, **p<0.05,*  ****p<0.01*

## Appendix 2 – Supplementary materials

### Appendix 2.1 – Representativeness of sample

Quality and Outcomes Framework, 2020-21

| Prevalence | OHFT CCGs | | | England CCGs | | |
| --- | --- | --- | --- | --- | --- | --- |
|  | Bath and North East Somerset, Swindon and Wiltshire | Buckinghamshire | Oxfordshire | Lower quartile | Upper quartile | Total |
| Dementia | 0.8 | 0.7 | 0.7 | 0.7 | 0.9 | 0.8 |
| Depression | 11.1 | 12.1 | 12.6 | 11.7 | 14.5 | 13.1 |
| Epilepsy | 0.8 | 0.7 | 0.7 | 0.8 | 0.9 | 0.9 |
| Learning disabilities | 0.5 | 0.4 | 0.4 | 0.5 | 0.6 | 0.6 |
| Mental Health | 0.8 | 0.8 | 0.8 | 0.8 | 1.0 | 0.9 |

Mental Health Bulletin Annual Report, 2019-20

| Service statistic |  | England NHS providers | | |
| --- | --- | --- | --- | --- |
|  | OHFT | Lower quartile | Upper quartile | Total |
| Number of people in contact with NHS funded providers | 59,935 | 3,965 | 51,180 | 2,878,636 |
| Number of people admitted to hospital | 1,720 | 898 | 2,061 | 104,536 |
| % people in contact admitted to hospital | 3.0% | 3.0% | 4.0% | 3.6% |
| Number of in-year bed days | 153,185 | 49,899 | 173,380 | 9,548,031 |
| Number of admissions | 1,550 | 961 | 2,308 | 123,632 |
| Number of discharges | 1,515 | 986 | 2,336 | 124,560 |
| Mean occupied bed days | 420 | 137 | 475 | 26,159 |
| Total care contacts | 325,165 | 25,505 | 445,095 | 26,608,986 |
| Total care contacts attendance rate | 83.0% | 74.0% | 89.0% | 80.6% |
| Early Intervention Team for Psychosis contacts | 9,065 | 8,038 | 25,273 | 1,007,735 |
| Early Intervention Team for Psychosis contact attendance rate | 86.0% | 77.0% | 88.5% | 82.7% |
| Proportion EIP referrals entering treatment within two weeks | 80.0% | 65.0% | 80.0% | 67.7% |

### Appendix 2.2 – Weekly totals descriptive statistics

| **Outcome** | | Mean | SD | N |
| --- | --- | --- | --- | --- |
| Outpatient and community referrals: | |  |  |  |
|  | Total referrals | 364.75 | 56.62 | 226 |
|  | First referrals | 95.84 | 18.52 | 122 |
|  | Early Intervention in Psychosis | 8.92 | 3.65 | 226 |
|  | Community Mental Health Teams | 49.38 | 18.21 | 226 |
|  | Crisis Teams | 30.49 | 7.96 | 226 |
|  | Psychiatric Liaison | 46.88 | 8.74 | 226 |
|  | Mean days to contact after referral | 262.38 | 116.27 | 226 |
|  | % referrals with contact within 30 days | 0.49 | 0.05 | 226 |
| Outpatient and community contacts: | |  |  |  |
|  | Total contacts | 2089.18 | 319.80 | 226 |
|  | First contacts | 104.46 | 16.86 | 122 |
|  | Face-to-face contacts | 1633.70 | 377.72 | 226 |
|  | Remote contacts | 446.89 | 480.06 | 226 |
|  | Early Intervention in Psychosis | 148.23 | 38.72 | 226 |
|  | Community Mental Health Teams | 677.07 | 109.81 | 226 |
|  | Crisis Teams | 38.31 | 10.12 | 226 |
|  | Psychiatric Liaison | 57.70 | 11.28 | 226 |
| Inpatient admissions: | |  |  |  |
|  | Total admissions | 15.17 | 3.99 | 226 |
|  | First admissions | 9.07 | 3.11 | 122 |
|  | Admissions to acute/PICU wards | 10.48 | 3.27 | 226 |
|  | % who were discharged in last 30 days | 0.07 | 0.07 | 222 |
|  | Total bed days | 1104.55 | 120.52 | 226 |
|  | Unique inpatients | 172.19 | 18.35 | 226 |
|  | Inpatient length of stay | 65.61 | 37.96 | 226 |
| HoNOS assessments: | |  |  |  |
|  | Total assessments | 195.71 | 31.66 | 226 |
|  | Initial assessments | 103.21 | 16.80 | 226 |
|  | Ongoing assessments | 66.26 | 17.16 | 226 |
|  | Discharge assessments | 26.24 | 7.50 | 226 |
| HoNOS scores: | |  |  |  |
|  | Mean total score | 11.51 | 0.66 | 226 |
| Overactive, aggressive, disruptive or agitated behaviour | |  |  |  |
|  | Any problems | 0.47 | 0.04 | 226 |
|  | Mean score | 0.83 | 0.10 | 226 |
| Non-accidental self-injury | |  |  |  |
|  | Any problems | 0.29 | 0.04 | 226 |
|  | Mean score | 0.57 | 0.09 | 226 |
| Problem-drinking or drug-taking | |  |  |  |
|  | Any problems | 0.24 | 0.03 | 226 |
|  | Mean score | 0.48 | 0.08 | 226 |
| Cognitive problems | |  |  |  |
|  | Any problems | 0.42 | 0.05 | 226 |
|  | Mean score | 0.83 | 0.11 | 226 |
| Physical illness or disability or disability problems | |  |  |  |
|  | Any problems | 0.49 | 0.04 | 226 |
|  | Mean score | 1.01 | 0.10 | 226 |
| Problems associated with hallucinations and delusions | |  |  |  |
|  | Any problems | 0.29 | 0.04 | 226 |
|  | Mean score | 0.59 | 0.09 | 226 |
| Problems with depressed mood | |  |  |  |
|  | Any problems | 0.78 | 0.03 | 226 |
|  | Mean score | 1.59 | 0.09 | 226 |
| Other mental and behavioural problems | |  |  |  |
|  | Any problems | 0.81 | 0.03 | 226 |
|  | Mean score | 1.87 | 0.10 | 226 |
| Problems with relationships: | |  |  |  |
|  | Any problems | 0.61 | 0.04 | 226 |
|  | Mean score | 1.19 | 0.12 | 226 |
| Problems with activities of daily living | |  |  |  |
|  | Any problems | 0.55 | 0.05 | 226 |
|  | Mean score | 1.07 | 0.12 | 226 |
| Problems with living conditions | |  |  |  |
|  | Any problems | 0.27 | 0.04 | 226 |
|  | Mean score | 0.49 | 0.08 | 226 |
| Problems with occupation and activities | |  |  |  |
|  | Any problems | 0.53 | 0.05 | 226 |
|  | Mean score | 1.01 | 0.12 | 226 |
| HoNOS repeated assessments (within 12 weeks) | |  |  |  |
|  | Mean score change | -1.34 | 1.75 | 226 |
|  | % with overall deterioration | 0.35 | 0.13 | 226 |
|  | % with overall improvement | 0.53 | 0.13 | 226 |

### Appendix 2.3 – Health of the Nation Outcome Scales (HoNOS)

The HoNOS sub-scales are:

- Overactive, aggressive, disruptive or agitated behaviour
- Non-accidental self-injury
- Problems with drinking or drug-taking
- Cognitive problems
- Physical illness or disability
- Problems associated with hallucinations or delusions
- Problems with depressed mood
- Other mental and behavioural problems (most commonly anxiety)
- Problems with relationships
- Problems with activities of daily living
- Problems with living conditions
- Problems with occupations and activities

## Appendix 3 - Methodology

### Appendix 3.1 - Correcting for censoring in duration outcomes

A number of outcome variables in our analysis are measures of duration, specifically length of stay, and waiting times. As with other variables, these are analysed not at a patient level but as a weekly mean, to allow for the use of interrupted time series analysis. Unlike other weekly summary variables (e.g. mean HoNOS scores or inpatient admission counts), the duration variables are impacted by the right-censoring of observations that remain “open” (i.e. not yet discharged or still waiting for a contact) at the time of the data extract. Weeks earlier in the sample period will contain full, uncensored observations while weeks at the end of the sample will contain more censored observations, biasing our estimate of the impact of COVID-19 downward. However, the durations recorded later in the sample do contain information about the mean length of stay, as relatively higher or lower levels of uncensored observations indicate relatively shorter or longer mean durations respectively.

For example, the equation shows how the observed mean length of stay for each week is calculated:

$${\hat{\mathrm{LoS}}}_{t}^{\mathrm{obs}}=\frac{1}{n_{t}}\Sigma_{i}\mathrm{LoS}_{i,t}^{\mathrm{obs}}$$

where  ${\hat{\mathrm{LoS}}}_{t}^{\mathrm{obs}}$ is the observed mean length of stay (not accounting for censoring) in week $t$, equal to the sum of observed lengths of stay for each individual admission $i$, divided by the total number of admissions $n$.

$${\hat{\mathrm{LoS}}}_{t}^{\mathrm{est}}=\frac{1}{n_{t}}\Sigma_{i}\left( \mathrm{LoS}_{i,t}^{\mathrm{obs}}+\left( C_{i}\times\int_{\mathrm{LoS}^{\mathrm{obs}}}^{\infty} S_{i}(t)dt \right) \right)$$

By contrast, the above equation shows how the estimated length of stay (correcting for censoring is calculated). In cases where the length of stay is censored ($C_{i}=1)$, the observed length of stay is added to the conditional expected survival time (from the point of censoring), and the mean of the combined variable is taken. In weeks where a greater proportion of admissions are censored, a greater proportion of the expected length of stay will be determined by expected conditional mean survival team. The estimate of true mean length of stay will be unbiased so long as the estimated survival function is accurate.

The survival function for each censored observation is estimated by fitting a parametric survival model to the full population of duration episodes (e.g. all inpatient stays). The functional form for each type of duration has been selected by visually inspecting goodness of fit of the survival function against common parametric survival functions (exponential, Weibull, normal, lognormal, log-logistic and gamma). In practice, all of the survival functions most closely followed a lognormal distribution, so the remaining imputed time for a censored duration is calculated with estimated log-mean and log-sd parameters.

An alternative approach to check the accuracy of effects found through changes in estimated length of stay is to analyse the proportion of observations exceeding a specific length threshold (e.g. 30 days). Since the data extract was taken at the end of August and our analysis only covers to the start of July, this does not restrict our sample. It is however possible that the mean duration could change without any impact around the 30 day threshold (e.g. due to a small increase in very long inpatient stays or outpatient waits) which would nonetheless be important to understanding service provision.

### Appendix 3.2 – Time series specification

Failing to account for serial correlation in the interrupted time series analysis of outcome variables has potential to bias the estimated impacts of interest. To account for this we followed a step-wise procedure for selecting Autoregressive Integrated Moving Average (ARIMA) models following Schaffer, Dobbins (1).

For each outcome variable, we first test for the presence of a unit-root using a Dickey-Fuller test (with a constant, a linear trend and 5 autoregressive lags). In practice, all the tests were able to reject the null hypothesis of a unit root, meaning all of the models used were ARMA rather than ARIMA.

Once the order of difference had been established (*d*=0), the residuals from an OLS specification of the ITS model were tested for autocorrelation and partial autocorrelation. An AR model of an order equal to the lowest order of statistically significant autocorrelation (Ljung-Box test rejects the null hypothesis of independent distribution at the level of $p<0.05$) was used as a starting point for the algorithmic selection of an ARIMA model. The algorithm would then test for stepwise improvement (as measured by AIC) on the starting model by adding and removing AR and MA terms (of no higher order than the highest order of serial correlation exhibited in the initial OLS estimates) until no improvement was possible. This specification of the form ARIMA(*p,* 0*, q*) where *p* is equal to the number of AR terms, 0 is the order of integration and *q* is the number of MA terms is shown next to the results of each outcome.

### Appendix 3.3 - Propensity score weighting

Variables measuring mean outcomes or durations for patients assessed, admitted or referred in a given week were influenced not only by the effects of COVID-19 but also by the composition of patients. Since our analysis is at the level of weekly aggregates, incorporating compositional measures (e.g. proportion of patients from deprived areas) directly into the regressions as independent variables would significantly reduce the degrees of freedom of our final models. Instead, we have used two adapted forms of propensity scores to minimise the bias of compositional changes on the estimated true impact of COVID-19.

For each time series of weekly patients (e.g. patients undergoing HoNOS assessments or patients referred to outpatient services) we estimated the following relationship:

$$\Pr\left( W_{t}\geq\mathrm{March}{23}^{\mathrm{rd}}, 2020 \right)=f(X_{t})$$

Where $W_{t}$ is the starting date of week $t$ and $X_{t}$ is a vector of summary statistics for week $t$, including the proportion of patients from an ethnic minority, who are female and with a specific recorded diagnosis (statistics of which for the whole sample are in Table 1). The relationship was estimated in each case using a logistic regression.

The estimated propensity score for each week (interpreted as the likelihood of a week appearing in the post-COVID period based on its composition of patients) is then used twice in the final modelling process:

- Each weekly observation is weighted *proportional* to its propensity score if it occurs before the COVID-19 period ($1/(1-pscore))$ and *inversely proportional* to its propensity score if it occurs after the COVID-19 cutoff ($1/pscore$). This upwardly weights “control” weeks that are compositionally similar to “treatment” weeks and downwardly weights less compositionally similar treatment weeks.
- The propensity score itself is included as an independent variable in the final ARIMA model specification, to control for any remaining impact of compositional changes on the outcome of interest.

None of these adjustments are used for variables that do not depend on compositional changes in patients, for example those which are simply measures of the volume of patients accessing a specific service.

### Appendix 3.4 - Normal approximation of count variables

A number of outcome variables are counts of a number of patients, for example the number of inpatient admissions in a given week. While it may be more appropriate to model these variables explicitly with count distributions (e.g. Poisson), this would have impacted our ability to conduct and test the ARIMA specification used consistently across all outcomes.

Since the normal distribution is an approximation for the binomial distribution at sufficiently high numbers, we tested count specifications (specifically negative binomial and Poisson regressions) of each outcome against equivalent OLS ITSA models (with no ARIMA specification). A variable was considered to not approximate the normal distribution and therefore excluded if the AIC for a count model was lower than for the OLS model, and the proportional probability of minimizing information loss ($\exp\left( \text{AIC}_{\text{count}}-\text{AIC}_{\text{OLS}} \right)/2)$ was less than 0.05, or a difference in AIC of around 6. For simplicity, outcome variables with a lower mean and minimum than another excluded variable were also excluded due to not approximating the normal distribution, and the opposite was done for variables with a higher mean and minimum that had not been excluded.

None of the outcome variables for the full population were excluded, with the largest difference in AIC for first inpatient admissions ($\text{AIC}_{\text{count}}-\text{AIC}_{\text{OLS}}=-4.27$ ). However for the sub-group analysis, a number of outcomes (including inpatient admissions, referrals and contacts with specific services, and types of HoNOS assessment) were excluded from the analysis as they did not pass the same test due to the lower sample sizes.

1. Schaffer AL, Dobbins TA, Pearson S-A. Interrupted time series analysis using autoregressive integrated moving average (ARIMA) models: a guide for evaluating large-scale health interventions. BMC medical research methodology. 2021;21(1):1-12.
